# SMYD3 Promotes Immune Evasion in Clear Cell Renal Cell Carcinoma via SREBP1‐Mediated Transactivation of CD47

**DOI:** 10.1002/advs.202404200

**Published:** 2025-06-23

**Authors:** Zhengfang Liu, Xiumei Zhao, Maolin Zang, Huiyang Yuan, Xin Qin, Xiaofeng Li, Shuo Zhao, Ruirong Tan, Keqiang Yan, Li Liu, Yidong Fan, Ning Zhang, Benkang Shi, Bo Han, Shouzhen Chen

**Affiliations:** ^1^ Thoracic Surgery Department Shandong Cancer Hospital and Institute Shandong First Medical University and Shandong Academy of Medical Science Jinan 250117 China; ^2^ Department of Urology Qilu Hospital of Shandong University Jinan 2500112 China; ^3^ Department of Pathology Qilu Hospital of Shandong University Jinan 250012 China; ^4^ Department of Hematology The Second Hospital of Shandong University Jinan 250033 China; ^5^ Department of Urology The First Affiliated Hospital of Shandong First Medical University & Shandong Provincial Qianfoshan Hospital Shandong Medicine and Health Key Laboratory of Organ Transplantation and Nephrosis Shandong Institute of Nephrology Jinan 250013 China; ^6^ Translational Chinese Medicine Key Laboratory of Sichuan Province Sichuan Institute for Translational Chinese Medicine Sichuan Academy of Chinese Medicine Sciences Chengdu 610042 China; ^7^ School of Nursing Beijing University of Chinese Medicine Beijing 100191 China; ^8^ Department of Breast Surgery General Surgery Qilu Hospital of Shandong University Jinan 250012 China; ^9^ Department of Pathology Peking University People's Hospital Beijing 100044 China

**Keywords:** clear cell renal cell carcinoma, immune evasion, SMYD3/SREBP1/CD47 signaling

## Abstract

Cancer cell‐intrinsic features (e.g., genetic aberrations and dysregulation of signaling pathways) play pivotal roles in orchestrating the composition and functional state of the immune landscape, which in turn impact tumor progression and response to immunotherapy. Here, it is discovered that dysregulation of cancer cell‐intrinsic SET and MYND domain‐containing protein 3 (SMYD3) leads to the orchestration of an immunosuppressive microenvironment and the impairment of responses to PD‐1 blockade by reprogramming the infiltration of immune cells in the tumor microenvironment of clear cell renal cell carcinoma (ccRCC). SMYD3 cooperates with Sp1 to transcriptionally promote sterol regulatory element‐binding protein 1 (SREBP1) expression by modifying H3‐K4 di‐/trimethylation and consequently activating the transcription of CD47. CD47, a bridge between innate and adaptive immunity, acts as the downstream effector molecule of the SMYD3 signal to promote the infiltration of T helper 2 (Th2) cells, protecting renal cancer cells from immune attack. In summary, the critical role of the cancer cell‐intrinsic SMYD3‐SREBP1‐CD47 axis is elucidated in regulating the immune microenvironment in ccRCC and provides a potential therapeutic strategy to manipulate the tumor immune milieu in favor of antitumor immunity.

## Graphical abstract

Cancer cell‐intrinsic SMYD3 orchestrates an immunosuppressive microenvironment and impairs the response to PD‐1 blockade by reprogramming immune cells landscape in the tumor microenvironment of clear cell renal cell carcinoma (ccRCC). The immunomodulatory function of SMYD3 is mediated via the SREBP1/CD47 pathway. In vivo, a combination of SMYD3 inhibitor and anti‐PD‐1 shows promise as an effective treatment for ccRCC.

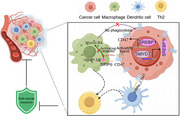

## 1 Introduction

Renal cell carcinoma (RCC) is one of the most common types of cancer in the urological system, with an increased incidence rate and more than 300 000 RCC‐related deaths worldwide in 2040.^[^ ^1^ ^]^ Clear cell renal cell carcinoma (ccRCC), the most common histological subtype of RCC,^[^ ^2^ ^]^ is characterized by inactivation of von Hippel‐Lindau (VHL) gene function, which leads to hypoxia inducible factor (HIF) signaling activity promotion and subsequent angiogenesis.^[^ ^3^ ^]^ Moreover, ccRCC stands out as one of the most common immune‐infiltrated tumors in pan‐cancer comparisons.^[^ ^4^ ^]^ Leveraging these features of ccRCC, the therapeutic landscape of ccRCC has undergone tremendous changes, and molecularly targeted agents, immune checkpoint inhibitors (ICIs) and their combination have been applied in the management of ccRCC beyond surgical resection over the past decade.^[^ ^5^ ^]^

It is well known that ccRCC is a tumor type that responds to immunotherapy.^[^ ^6^ ^]^ Since the 1990s, prior to the advent of molecularly targeted therapy, cytokine‐based immunotherapy has been the basic treatment for advanced renal cancer.^[^ ^7^ ^]^ In recent years, the use of modern immunotherapy, represented by ICIs that block the T‐cell inhibitory receptors PD‐1/PD‐L1 or CTLA4, has increased progression‐free survival (PFS) in both treatment‐naïve and pretreated patients with RCC.^[^ ^8^ ^]^ However, the objective response rates (ORRs) are only 25% in patients treated with nivolumab (a PD‐1 blocker) and 13% in patients treated with ipilimumab (a CTLA‐4 blocker).^[^ ^8^, ^9^ ^]^ Considering that the rate of positive PD‐L1 expression in RCC is as high as 40–61%, the current objective response rate fails to meet expectations.^[^ ^10^ ^]^ In addition, almost all patients either have primary resistance to ICIs or acquire resistance after an initial response.^[^ ^6^ ^]^ Mounting evidence reveals that the crosstalk between cancer cells and immune cells plays an important role in regulating the composition and functional state of the immune landscape.^[^ ^11^ ^]^ With this in mind, further research into the mechanisms driving the immunoregulatory function of renal cancer cells is needed to discover biomarkers that can predict the response to ICIs, and new treatment strategies for patients with ccRCC are needed.

SET and MYND domain‐containing protein 3 (SMYD3), which belongs to the histone lysine methyltransferase (KMT) family, regulates chromatin accessibility and gene expression via the methylation of histone or nonhistone substrates.^[^ ^12^ ^]^ Dysregulation of KMTs can lead to a variety of diseases, including cancers.^[^ ^13^ ^]^ Our previous study findings confirmed that upregulated SMYD3 expression leads to the promotion of tumorigenesis and progression in prostate cancer and renal cell carcinoma,^[^ ^14^ ^]^ but these studies focused only on the effect of SMYD3 on the cancer cells themselves, and whether and how cancer cell‐intrinsic SMYD3 impacts the crosstalk between RCC cells and immune cells are unknown.

In this study, we performed bioinformatic analysis and single‐cell RNA sequencing (scRNA‐seq), and we determined that SMYD3 promotes the infiltration of T helper 2 (Th2) cells and knockdown of SMYD3 strengthens the anti‐PD‐1 response in ccRCC cells. Importantly, we demonstrated that SMYD3 exerts an immunosuppressive effect by epigenetically promoting H3‐K4 di‐/trimethylation in the sterol regulatory element‐binding protein 1 (SREBP1) promoter region, and activating the transcription of SREBP1. Consequently, SREBP1 recognizes and binds sterol regulatory elements (SREs) in the promoter of CD47 to upregulate CD47 expression in ccRCC cells. CD47 is mediated through its interaction with the SIRPα receptor on myeloid cells, to create an inhibitory signaling pathway that enables tumor cells to evade immune surveillance. Together, these results indicate a critical role of the SMYD3‐SREBP1‐CD47 axis in immune invasion, thereby providing insights for future therapeutic approaches that could improve the management of patients with ccRCC.

## 2 Results

### 2.1 Increased SMYD3 Expression is Positively Related to Poor Immune Infiltration and Decreased Survival in Patients with ccRCC

SMYD3 is a histone methyltransferase that contributes to the development and progression of multiple types of cancers, and we analyzed SMYD3 mRNA expression and protein expression in renal tumors and adjacent normal tissues from both our data and publicly available gene expression profile data (e.g., the GEO and Oncomine databases). SMYD3 expression was upregulated at both the mRNA and protein levels (Figure S1A–D, Supporting Information), which was in line with the findings of previous studies of other types of tumors.^[^ ^12^ ^]^ We established stable Smyd3 knockdown RENCA cells, and Smyd3 knockdown impaired tumor growth in an orthotopic syngeneic mouse model in vivo (Figure S2A–D, Supporting Information). Additionally, we used Kaplan‐Meier plotter to perform Kaplan‐Meier analysis of the publicly available data and tissue microarray (TMA) data, which revealed that patients with high SMYD3 expression had shorter overall survival (OS) and recurrence‐free survival (RFS) (Figure S1E–G, Supporting Information).

**Figure 1:**
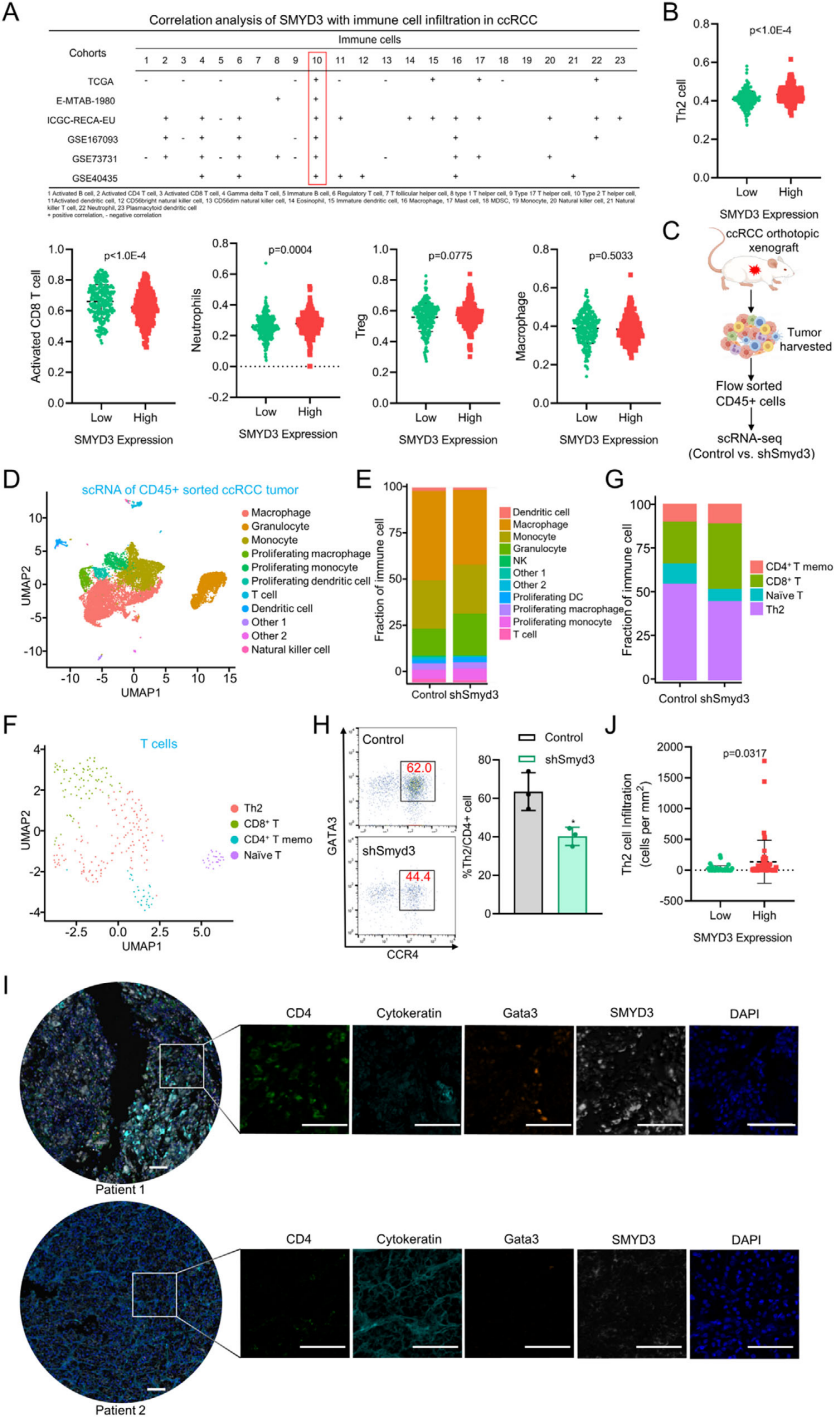
Upregulated SMYD3 expression is positively related to poor immune infiltration in ccRCC: A) Table of immune cell infiltration analysis results based on SMYD3 mRNA expression levels determined by ssGSEA of ccRCC cohorts (TCGA, E‐MATAB‐1980, ICGC‐RECA‐EU, GSE167093, GSE73731, and GSE40435). 1 Activated B cell, 2 Activated CD4 T cell, 3 Activated CD8 T cells, 4 Gamma delta T cell, 5 Immature B cell, 6 Regulatory T cell, 7 T follicular helper cell, 8 type 1 T helper cell, 9 Type 17 T helper cell, 10 Type 2 T helper cell, 11Activated dendritic cell, 12 CD56bright natural killer cell, 13 CD56dim natural killer cell, 14 Eosinophil, 15 Immature dendritic cell, 16 Macrophage, 17 Mast cell, 18 MDSC, 19 Monocyte, 20 Natural killer cell, 21 Natural killer T cell, 22 Neutrophil, 23 Plasmacytoid dendritic cell. B) Correlation analysis of SMYD3 expression with Th2 cell, activated CD8 T cell, neutrophils, Treg, and macrophage infiltration in the TCGA ccRCC dataset (*n* = 530). Student's *t*‐test. C) Schematic showing the experimental strategy for single‐cell RNA sequencing (scRNA‐seq) of the indicated RENCA tumor‐bearing mice. D) Uniform manifold approximation and projection (UMAP) plot showing eleven immune cell subsets identified by scRNA‐seq. E) Bar graph showing the proportions of immune cell subsets in ccRCC samples. F) UMAP plot of T cells showing 4 major clusters identified by scRNA‐seq. G) Bar graph showing the proportions of T‐cell subsets in ccRCC samples. H) Flow cytometry gating and frequency of Th2 cells (GATA3^+^CCR4^+^CD4^+^) among total CD4^+^ T cells in RENCA tumors stably expressing SMYD3 shRNA or control shRNA (*n* = 3). *p < 0.05, Student's *t*‐test. I) Representative multiplex immunofluorescence images of ccRCC tumor samples (details given in Table S5, Supporting Information) displaying 2 TMA cores (Patient 1 and Patient 2) after multispectral imaging and enlarged subsections of the core showing each of the individual markers in the composite image after spectral unmixing. Markers: CD4 (Opal 520, pseudocolored green), cytokeratin (Opal 480 pseudocolored cyan), Gata3 (Opal 620, pseudocolored orange), SMYD3 (Opal 780, pseudocolored white), and DAPI was used as a nuclear marker (pseudocolored blue). Scale bars: 100 µm. J) Quantification of Th2 cell infiltration in the TMA per tumor area (mm^2^) in patients with ccRCC (*n* = 45). Student's *t*‐test. Data are presented as mean ± SEM.

The interactions between tumor cells and the microenvironment are key in carcinogenesis and tumor progression, and immune cells play pivotal roles in the tumor microenvironment (TME).^[^ ^15^ ^]^ RCC stands out as one of the most common immune‐infiltrated tumors in pan‐cancer comparisons.^[^ ^16^ ^]^ We subsequently assessed the role of cancer cell‐intrinsic SMYD3 in the tumor immune microenvironment (TIME) of ccRCC. We first investigated the effect of SMYD3 on infiltrating immune cell type through single sample Gene Set Enrichment Analysis (ssGSEA) on the basis of RNA‐seq information from ccRCC datasets (TCGA, E‐MATAB‐1980, ICGC‐RECA‐EU, GSE167093, GSE73731 and GSE40435), which include more than 100 cases of ccRCC, with the exception of ICGC‐RENCA‐EU (*n* = 91). We found that increased expression of SMYD3 was associated with decreased infiltration of T helper 17 (Th17) cells and activated CD8^+^ T cells, as well as increased infiltration of regulatory T cells (Tregs), neutrophil, especially Th2 cells (**Figure** **1**A,B; Figure S3A–F, Supporting Information). Moreover, the finding that high SMYD3 expression is associated with high Th2 cell infiltration was validated in all six large ccRCC datasets, and Th2 cells might be associated with a negative outcome by promoting immune evasion in ccRCC.^[^ ^4^ ^]^

### 2.2 Landscape of the Cell Composition Regulated by Smyd3 in the TIME According to scRNA‐Seq Data

To gain more insight into the immune landscape changes induced by SMYD3 in ccRCC, we established a stable Smyd3‐knockdown RENCA cell line by short hairpin RNA (shRNA) lentivirus and generated scRNA‐seq profiles of FACS‐sorted CD45^+^ immune cells isolated from tumors in orthotopic syngeneic renal cell carcinoma BALB/c mice using 10× Genomics sequencing (Figure 1C). After strict quality control, 7592 cells in control group and 8123 cells in shSmyd3 group were identified.

Unsupervised clustering analysis was performed using Seurat. Clusters were defined on the basis of variable gene expression and canonical markers after normalization. Eleven distinct cell types were identified in the cells analyzed by scRNA‐seq in both shSmyd3 and control tumors and visualized with uniform manifold approximation and projection (UMAP) (Figure 1D). The eleven distinct cell types included macrophages, granulocytes, monocytes, proliferating macrophages, proliferating monocytes, proliferating dendritic cells, T cells, dendritic cells, natural killer cells, other 1 and other 2 cells. The majority of immune cells identified were macrophages, followed by monocytes and granulocytes. Macrophages were enriched in the control group (Figure 1D,E). Granulocyte subgroup analysis showed that the granulocytes in our study were primarily composed of neutrophils (Figure S4A, Supporting Information). Neutrophils were further classified into N1 (anti‐tumor), N2 (pro‐tumor), and undifferentiated neutrophils, while macrophages were divided into M1, M2, and undifferentiated macrophages.^[^ ^17^ ^]^ (Figure S4B,D, Supporting Information). The frequency of neutrophils and macrophages from each cluster did not differ between control and shSmyd3 groups (Figure S4C,E, Supporting Information). However, knockdown Smyd3 expression of tumor cells could impact the transcriptional changes in tumor‐infiltrating neutrophils and macrophages, especially the expression of Cebpb (Figure S4F, Supporting Information), which encodes a crucial transcription factor for emergency granulopoiesis and monocyte/macrophage gene regulation,^[^ ^18^ ^]^ and is well known for its role in immunosuppression.^[^ ^19^ ^]^

Given that T cells play a vital role in regulating TME, we next explored whether Smyd3 regulates the T‐cell subpopulation, we performed subpopulation analysis of the T‐cell (Figure 1F). High infiltration of Th2 cells can cause epigenetic reprogramming of tumor cells and suppress breast tumorigenesis and Th2 cells trigger an inflammatory immune response to slow tumor growth.^[^ ^20^ ^]^ However, recent studies have demonstrated that T helper 2 (Th2) cells are positively correlated with immunosuppressive environment in ccRCC.^[^ ^4^ ^]^ Moreover, Th2 cells can stimulate cancer cell‐intrinsic MYC transcriptional upregulation to drive glycolysis, foster an immunosuppressive microenvironment, and aid in tumor progression.^[^ ^21^ ^]^ The results of T‐cell subpopulation analysis revealed that Th2 cells were enriched in the control group (Figure 1G). Moreover, our flow cytometry data confirmed that Smyd3 knockdown could reduce the infiltration of Th2 (CD4^+^GATA3^+^CCR4^+^) cells, which is consistent with the results of scRNA‐seq (Figure 1H).

To further confirm the clinical relevance of our findings concerning the immunophenotype of ccRCC, we performed multiplex immunofluorescence assays with tissue microarrays (TMAs) of human ccRCC. In line with the results of ssGSEA and scRNA‐seq, upregulated SMYD3 expression was associated with high infiltration of Th2 cells in ccRCC (Figure 1I,J). Additionally, increased numbers of Th2 cells were found in ccRCC tissues compared with normal tissues (Figure S5A,B, Supporting Information). Taken together, these data demonstrated that high levels of SMYD3 are correlated with poor outcomes and are associated with increased infiltration of Th2 cells in the ccRCC TIME.

### 2.3 SMYD3 Deficiency in ccRCC Cells Strengthens the Anti‐PD‐1 Response

**Figure 2:**
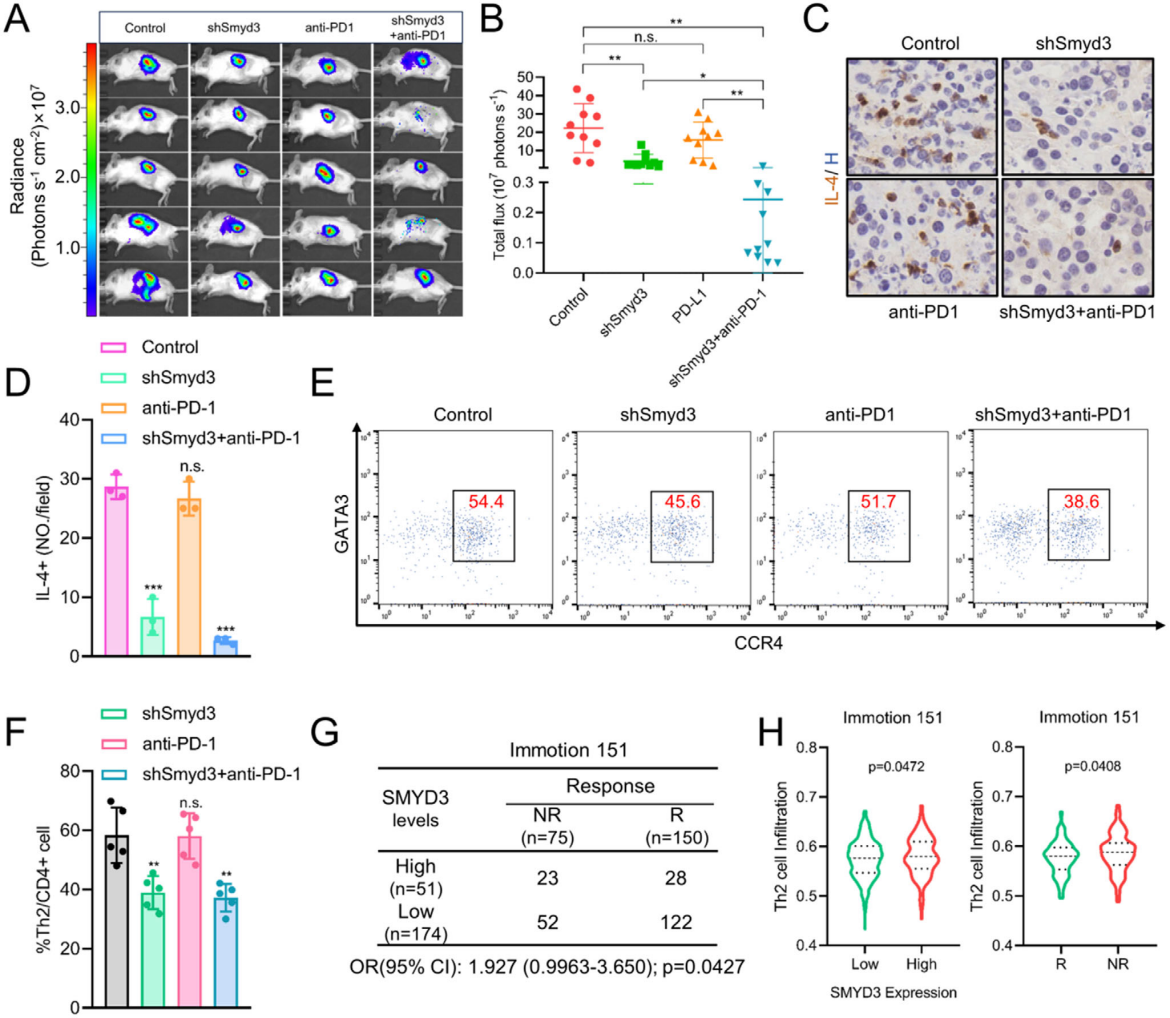
SMYD3 knockdown in ccRCC cells reduces Th2 cell infiltration and strengthens anti‐PD‐1 effects: A) Representative bioluminescence images of tumors in BALB/c mice orthotopically engrafted with control or SMYD3‐knockdown RENCA tumors treated with either isotype IgG or anti‐PD‐1 antibody. B) Burden of control and SMYD3‐knockdown RENCA tumors treated with isotype IgG or anti‐PD‐1 antibody as measured by bioluminescence (*n* = 10). n.s., non‐significant, *p < 0.05, **p < 0.01, one‐way ANOVA test. C) Representative images of orthotopic syngeneic mouse tumors stained for IL‐4. D) Quantification of IHC staining for IL‐4 (*n* = 3). n.s., non‐significant, ***p < 0.001, one‐way ANOVA test. E) Flow cytometry gating and frequency of Th2 cells among total CD4^+^ T cells in RENCA tumors treated with either isotype IgG or anti‐PD‐1 antibody. F) Frequencies of Th2 cells among total CD4^+^ T cells in RENCA tumors treated with either isotype IgG or anti‐PD‐1 antibody (*n* = 5). n.s., non‐significant, **p < 0.01, one‐way ANOVA test. G) Correlation analysis of SMYD3 expression and ICI response in ccRCC patients from the IMmotion151 cohort. R: response, NR: non‐response. Chi‐square test. H) Th2 cell infiltration analysis by ssGSEA in the IMmotion151 ccRCC cohort based on SMYD3 expression and ICI response (R, *n* = 150; NR, *n* = 75). R: response, NR: non‐response. Student's *t*‐test. Data are presented as mean ± SEM.

These results suggest that SMYD3 plays an important role in regulating immune cell infiltration in the TME of ccRCC. To further explore the role of SMYD3 in regulating ICI efficacy in vivo, we used a stable Smyd3‐knockdown RENCA cell line and constructed an orthotopic syngeneic mouse model in BALB/c mice. Smyd3 expression downregulation restored sensitivity to anti‐PD‐1 therapy and decreased luminescence (**Figure** **2**A,B). Moreover, Smyd3 knockdown reduced the infiltration of Th2 cells into the TME of RENCA tumors, which was consistent with the above results (Figure 2C–F). Furthermore, analysis of data from the RCC clinical trial IMmotion151^[^ ^22^ ^]^ revealed that high SMYD3 expression was associated with a lower ICI response rate (OR: 1.927; 95% CI: 0.9963–3.650), and the non‐responsive group had greater infiltration of Th2 cells than the responsive group did (Figure 2G,H). Moreover, SMYD3 expression is also positively correlated with Th2 cell infiltration in IMmotion151 (Figure 2H).

### 2.4 CD47 is the Inhibitory Checkpoint Target of SMYD3 that Mediates Immune Evasion in ccRCC

**Figure 3:**
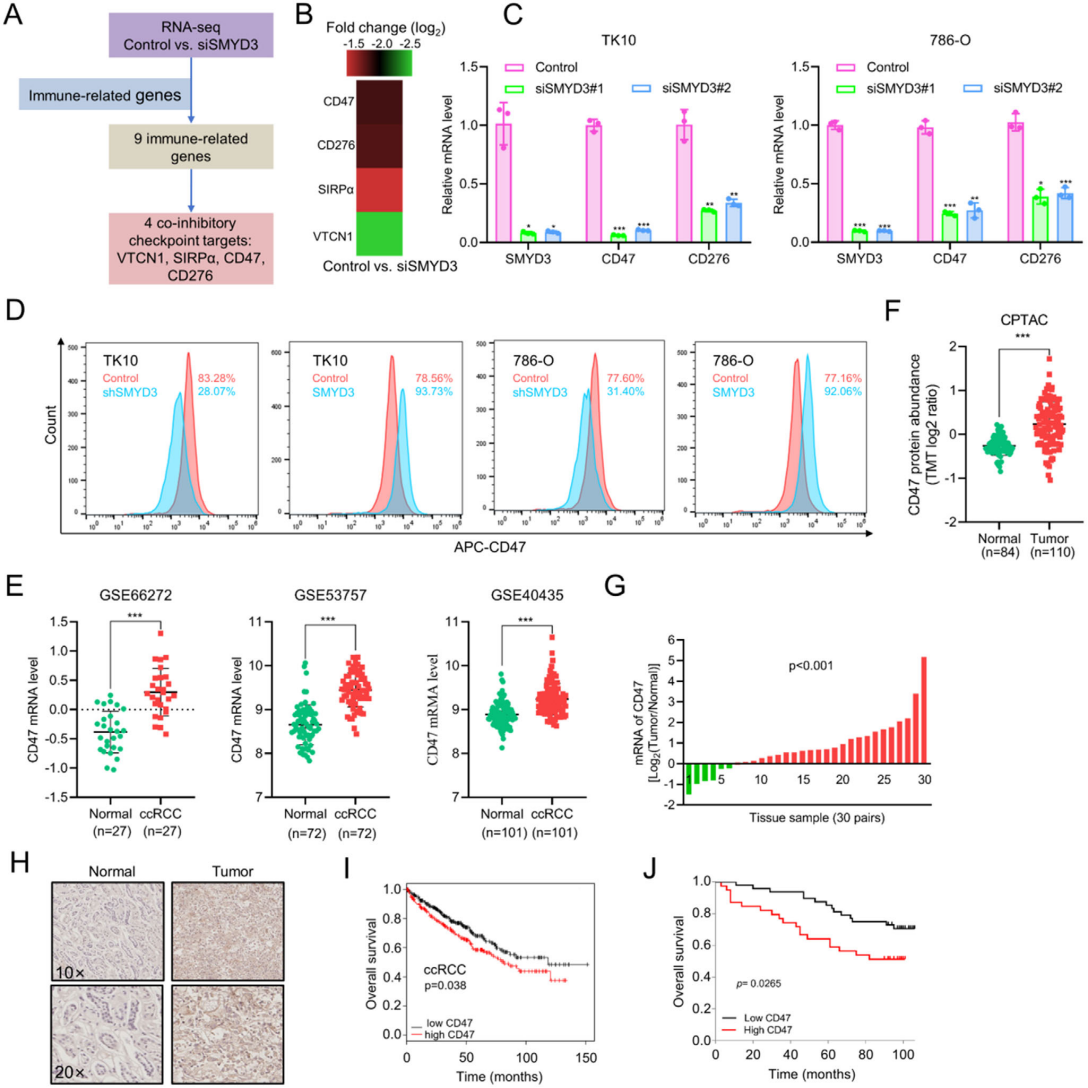
Upregulated CD47 expression is the inhibitory checkpoint target of SMYD3 and is associated with poor overall survival in patients with ccRCC: A) Schematic illustration of immune effector gene analysis affected by SMYD3 in ccRCC cells. B) Heatmap of the expression of these four immune inhibitory checkpoints upon SMYD3 knockdown according to the mRNA sequencing data. C) Changes in the mRNA expression levels of CD47 and CD276 upon SMYD3 knockdown in TK10 and 786‐O cells, as determined by qPCR (*n* = 3). *p < 0.05, **p < 0.01, ***p < 0.001, Student's *t*‐test. D) CD47 expression on the cell surface was determined by flow cytometry in ccRCC cells stably expressing SMYD3 shRNA or overexpressing SMYD3. E) CD47 transcript expression in multiple renal cancer studies from the GEO database. ***p < 0.001, Student's *t*‐test. F) CD47 protein expression in ccRCC patients from the CPTAC database. ***p < 0.001, Student's *t*‐test. G) mRNA expression of CD47 in renal tumor tissues and paired normal tissues (*n* = 30 pairs). Student's *t*‐test. H) Representative IHC staining images of CD47 expression at the protein level in tumors compared with normal tissues from the TMA cohort. I) Kaplan–Meier survival analysis of overall survival according to CD47 mRNA levels in patients with ccRCC from the KM‐plotter database. J) Kaplan–Meier plots comparing the OS of ccRCC patients according to CD47 protein abundance in the TMA cohort. Log‐rank test. Data are presented as mean ± SEM.

Our observations suggest that there is a clinical relationship between cancer cell‐intrinsic SMYD3 and the inhibitory immune microenvironment of ccRCC. However, the mechanism underlying SMYD3‐mediated crosstalk between cancer cells and immune cells and the downstream immune effector molecules of SMYD3 signaling remains unknown. To address these gaps in knowledge, we performed RNA sequencing of the ccRCC cell line TK10, which revealed 6070 deregulated genes upon SMYD3 knockdown by small interfering RNA (siRNA). Among these differentially expressed genes (DEGs), 3060 were downregulated, and 3010 were upregulated when si‐ctrl and si‐SYMD3 cells were compared (Table S1, Supporting Information). Next, we identified 9 immune‐related DEGs that fulfilled two criteria: (1) they were significantly deregulated by SMYSD3 in TK10 cells, and (2) they were included in an immune‐related gene set (Table S2, Supporting Information).^[^ ^23^ ^]^ Among these 9 immune‐related genes, 4 (CD276, CD47, SIRPα, and VTCN1) are inhibitory checkpoint targets that mediate immune evasion and might be regulated by SMYD3 in ccRCC (**Figure** **3**A). The fold changes in the expression of these 4 genes upon SMYD3 knockdown determined by RNA sequencing analysis are shown in Figure 3B. After further investigation of the expression profiles of ccRCC in TCGA data, VTCN1 was excluded from the analysis, as VTCN1 expression was lower in tumor tissues than in normal tissues (Figure S6A, Supporting Information). SIRPα is a transmembrane protein that binds to the extracellular Ig‐domain of CD47, and is expressed on myeloid cells, especially macrophages and dendritic cells, where it regulates phagocytosis and mediates the “don't eat me” signal upon binding to its ligand CD47 on tumor cells; the activity of the CD47/ SIRPα axis impairs innate and adaptative immunity.^[^ ^24^ ^]^ We subsequently performed stable knockdown of SMYD3 in TK10 and 786‐O cells using shRNA lentivirus and RT‐qPCR to verify the alterations in the expression levels of CD276 and CD47 (Figure 3C). The expression level of CD47 changed more significantly than that of CD276 upon SMYD3 knockdown. Because surface expression of CD47 is required for its anti‐phagocytic capacity, we then performed flow cytometry. SMYD3 knockdown decreased while SMYD3 overexpression increased CD47 cell‐surface expression in TK10 and 786‐O cells (Figure 3D). Taken together, these results suggest that CD47 is a downstream effector molecule of SMYD3 involved in promoting immune evasion in ccRCC.

### 2.5 CD47 is Upregulated and Associated with Poor Survival in Patients with ccRCC

We hypothesized that, as an immune effector molecule of SMYD3, CD47 would also have an elevated expression level in ccRCC. To test this hypothesis, we first evaluated CD47 expression in ccRCC publicly available gene expression profile data (GEO and oncomine) and found that CD47 mRNA expression was elevated in tumor tissues compared with normal tissues (Figure 3E; Figure S6B, Supporting Information). To verify whether the protein level of CD47 was consistent with the mRNA level in ccRCC, we analyzed CD47 protein expression in data from the public proteomic database CPTAC. The analysis revealed that the protein level of CD47 was significantly elevated in tumor tissues compared with normal tissues (Figure 3F). Furthermore, a similar expression pattern for CD47 was found in our cohort of 30 ccRCC tumors and matched normal tissues (Figure 3G). Additionally, the expression pattern of CD47 in the TMA measured by IHC staining was similar to the above findings (Figure 3H). We subsequently used Kaplan‐Meier plotter to perform Kaplan‐Meier analysis of the publicly available data to determine whether CD47 expression affects the outcomes of patients with ccRCC. As shown in Figure 3I, patients with higher CD47 expression in ccRCC had shorter OS than patients with lower expression of CD47. CD47 expression also had a negative effect on relapse‐free survival (RFS) in patients with kidney renal papillary cell carcinoma (KIRP) (Figure S6C, Supporting Information). Moreover, patients with upregulated CD47 expression were demonstrated to have shorter OS times on the basis of clinical data from the TMA (Figure 3J).

### 2.6 CD47 Deficiency Impairs Tumor Growth and the Infiltration of Th2 Cells in ccRCC

**Figure 4:**
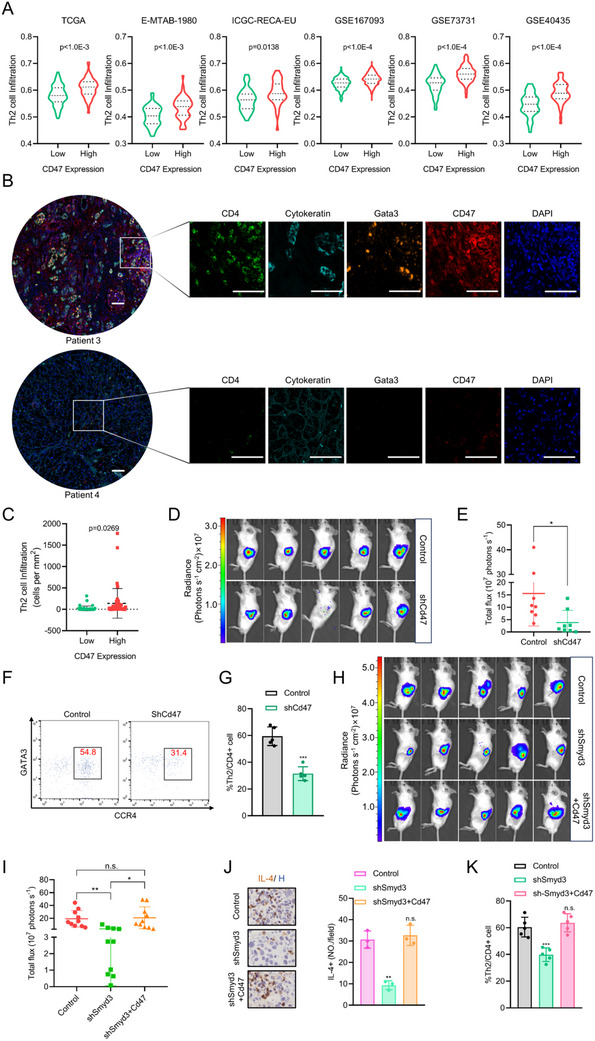
CD47 promotes Th2 cell infiltration in the TME of ccRCC: A) Th2 cell infiltration analysis on the basis of CD47 mRNA levels by ssGSEA in ccRCC cohorts (TCGA, E‐MATAB‐1980, ICGC‐RECA‐EU, GSE167093, GSE73731, and GSE40435). Student's *t*‐test. B) Representative multiplex immunofluorescence images of ccRCC samples (details given in Table S5) displaying 2 TMA cores (Patient 3(high expression of CD47) and Patient 4 (low expression of CD47)) after multispectral imaging and enlarged subsections of the core showing each of the individual markers in the composite image after spectral unmixing. The markers used were as follows: CD4 (Opal 520, pseudocolored green), cytokeratin (Opal 480, pseudocolored cyan), Gata3 (Opal 620, pseudocolored orange), CD47 (Opal 690, pseudocolored red), and DAPI as a nuclear marker (pseudocolored blue). Scale bars: 100 µm. C) Quantification of Th2 cell infiltration in the TMA per tumor area (mm^2^) in patients with ccRCC (*n* = 45). Student's *t*‐test. D) Representative bioluminescence images of tumors in BALB/c mice orthotopically engrafted with control or CD47‐knockdown RENCA tumors. E) Burden of control and CD47‐knockdown RENCA tumors as measured by bioluminescence (*n* = 8). *p < 0.05, Student's *t*‐test. F) Flow cytometry gating and frequency of Th2 cells among total CD4^+^ T cells in RENCA tumors with stable expression of CD47 shRNA or control shRNA. G) Frequencies of Th2 cells among total CD4^+^ T cells in RENCA tumors with stable expression of CD47 shRNA or control shRNA (*n* = 5). ***p < 0.001, Student's *t*‐test. H) Representative bioluminescence images of tumors in BALB/c mice orthotopically engrafted with control, Smyd3‐knockdown, and rescued Cd47‐overexpressing RENCA tumors. I) The burden of control, Smyd3‐knockdown, and rescued Cd47‐overexpression RENCA tumors as measured by bioluminescence (*n* = 10). n.s., non‐significant, **p < 0.01, one‐way ANOVA test. J) Representative images of orthotopic syngeneic mouse tumors stained for IL‐4 and quantification of IHC staining for IL‐4 (*n* = 3). n.s., non‐significant, ***p < 0.001, one‐way ANOVA test. K) Frequencies of Th2 cells among total CD4+ T cells in RENCA tumors in indicated groups (*n* = 5). n.s., non‐significant, **p < 0.01, ***p < 0.001, one‐way ANOVA test. Data are presented as mean ± SEM.

Given that CD47 is the downstream effector molecule of SMYD3 involved in orchestrating the inhibitory immune microenvironment in ccRCC, we explored the correlation between CD47 mRNA expression and Th2 cells in ccRCC. We found that CD47 expression was positively correlated with the gene set of Th2 cells in publicly available ccRCC cohorts (TCGA, E‐MTAB‐1980, ICGC‐RECA‐EU, GSE167093, GSE73731 and GSE40435) (**Figure** **4**A). Multiple immunofluorescence assays of TMAs were conducted to further evaluate the correlation between CD47 protein expression and Th2 cell infiltration. The results confirmed that a high CD47 expression level was positively correlated with Th2 cell infiltration (Figure 4B,C). Given that SMYD3 regulates CD47 expression, we next explored the effects of CD47 on tumor growth and the infiltration of Th2 cells. Knockdown of Cd47 impaired tumor growth in an orthotopic syngeneic mouse model, and less infiltration of Th2 cells into tumors was observed in Cd47‐knockdown RENCA tumors, as determined by flow cytometry (Figure 4D–G). To further explore whether SMYD3 promoted the infiltration of Th2 cells via CD47 in ccRCC, we overexpressed Cd47 in stable Smyd3‐knockdown RENCA cells to investigate whether the effects of Smyd3 knockdown could be eliminated upon Cd47 overexpression. Ectopic expression of Cd47 effectively reversed Smyd3 knockdown‐mediated tumor growth inhibition in an orthotopic syngeneic mouse model (Figure 4H,I). Additionally, immunohistochemical (IHC) staining revealed that ectopic expression of Cd47 abrogated the decreased IL‐4 protein level induced by Smyd3 knockdown (Figure 4J). Moreover, the decreased infiltration of Th2 cells caused by Smyd3 knockdown was abolished by Cd47 overexpression (Figure 4K).

### 2.7 SREBP1 Acts as the Pivotal Downstream Transcription Factor of SMYD3 to Promote Immune Evasion in ccRCC

**Figure 5:**
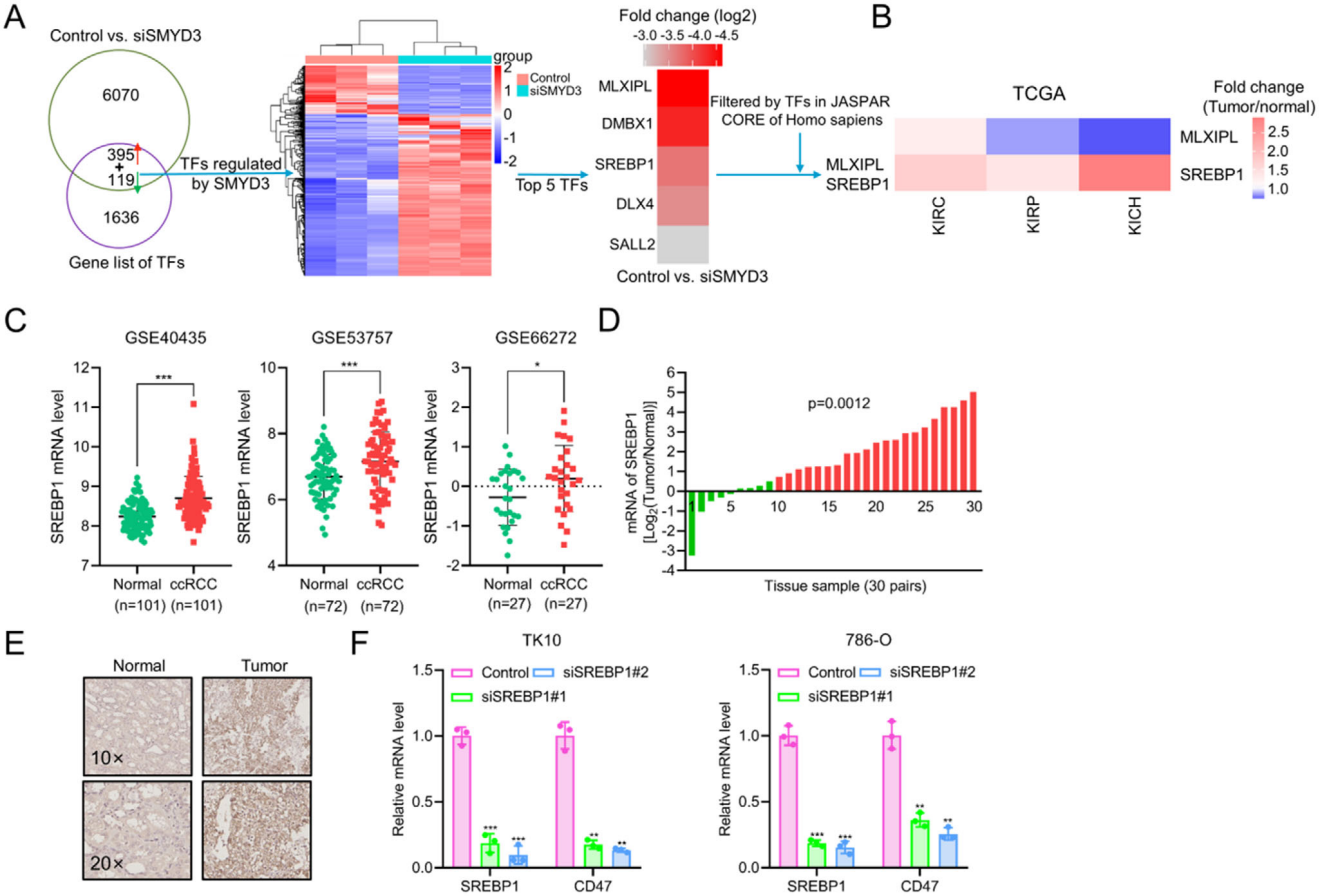
SREBP1 acts as the pivotal downstream transcription factor of SMYD3 to promote immune evasion in ccRCC: A) Schematic illustration of the effects of SMYD3 on transcription factors in ccRCC cells. B) Dysregulation of these two TFs (SREBP1 and MLXIPL) in 3 histological subtypes of RCC from TCGA. KIRC: kidney renal clear cell carcinoma; KIRP: kidney renal papillary cell carcinoma; KICH: kidney chromophobe. C) SREBP1 transcript expression in multiple renal cancer studies from the GEO database. *p < 0.05, ***p < 0.001, Student's *t*‐test. D) SREBP1 mRNA expression in renal tumor tissues and paired normal tissues (*n* = 30 pairs). Student's *t*‐test. E) Representative IHC images of SREBP1 expression at the protein level in tumors compared with normal tissues from the TMA cohort. F) Changes in the mRNA expression level of CD47 upon SREBP1 knockdown in TK10 and 786‐O cells, as determined by qPCR (*n* = 3). Data are presented as mean ± SEM of three experiments. **p < 0.01, ***p < 0.001, Student's *t*‐test. Data are presented as mean ± SEM.

We then explored the underlying mechanism by which SMYD3 regulates the expression of CD47 in ccRCC. Previous studies have revealed that SMYD3 mainly regulates downstream gene expression at the transcription level by recognizing and binding to the 5′‐CCCTCC‐3′ motif in the promoters of its target genes.^[^ ^14^ ^]^ To test whether CD47 is a direct transcriptional target of SMYD3, we screened the nucleotide sequence of the promoter region (2000 bp upstream of the transcription start site) of CD47, but we found no binding sites for SYMD3. Therefore, we speculated that other genes mediate the regulation of CD47 by SMYD3. Transcription factors (TFs) are the most important mediators in the regulation of gene expression involved in cellular physiological and pathological activities.^[^ ^25^ ^]^ We analyzed the RNA‐sequencing data of TK10 cells upon SMYD3 knockdown and first identified 119 SMYD3‐upregulated genes that fulfilled two criteria: (1) the expression of these genes was significantly upregulated by SMYD3 in TK10 cells and (2) a gene set of transcription factors was included (Table S3, Supporting Information).^[^ ^26^ ^]^ We then screened the top 5 transcription factors (MLXIPL, DMBX1, SREBP1, DLX4, and SALL2) with the most significant changes in expression upon SMYD3 knockdown for follow‐up analysis. Further investigation of the transcription factors of *Homo sapiens* listed in JASPAR CORE (http://jaspar.genereg.net/) pinpointed MLXIPL and SREBP1 as the functional candidate transcription factors regulated by SMYD3 in ccRCC (**Figure** **5**A).

To evaluate the potential roles of these two transcription factors in RCC tumorigenesis, we analyzed their mRNA expression in tumor and normal samples in the TCGA dataset. SREBP1 was the most commonly upregulated TF among the three types of RCC (Figure 5B). To further confirm the clinical relevance of SREBP1, we analyzed SREBP1 expression in other ccRCC clinical cohorts from the GEO and Oncomine databases. We found that SREBP1 expression was upregulated in tumor tissues compared with matched normal tissues (Figure 5C; Figure S7A, Supporting Information). Moreover, the mRNA expression pattern of SREBP1 in our cohort, which included 30 patients with ccRCC, was consistent with that observed in the above public datasets (Figure 5D). In addition to evaluating the expression of SREBP1 at the mRNA level, we also assessed the protein level in the TMA via IHC assays, and the data confirmed that the expression of SREBP1 was significantly elevated in tumor tissues compared with normal tissues (Figure 5E). To explore whether SREBP1 could regulate the expression of CD47, we performed RT‐qPCR assays, and the results demonstrated that SREBP1 could regulate the expression of CD47 at the transcription level (Figure 5F).

These results indicate that SREBP1 is a mediator TF in the regulation of CD47 by SMYD3, indicating the potential role of SREBP1 in the immunosuppressive microenvironment of ccRCC. We then performed ssGSEA of ccRCC data in the TCGA database to identify the infiltration of distinct immune cell subsets affected by SREBP1, and the results indicated that increased expression of SREBP1 was associated with decreased infiltration of natural killer cells and Th17 cells but increased infiltration of Th2 cells (Figure S7B, Supporting Information). As mentioned above, natural killer cells and Th17 cells constitute the antitumor immune microenvironment in ccRCC, and Th2 cells promote immune evasion. Taken together, these results suggest that SREBP1 regulates the expression of CD47 to mediate the immunosuppressive effect caused by upregulated SMYD3 expression in ccRCC.

### 2.8 SMYD3 Directly Transcriptionally Regulates the Expression of SREBP1 in ccRCC

**Figure 6:**
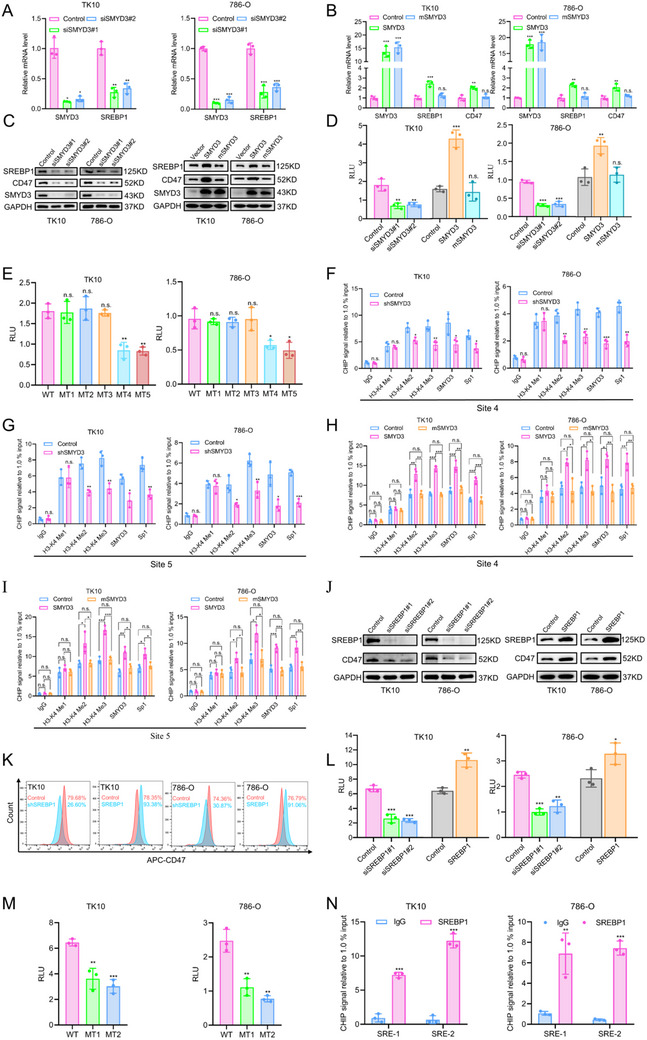
SMYD3 epigenetically activates the transcription of SREBP1, and CD47 is a transcriptional target of SREBP1 in ccRCC cells: A) Changes in SREBP1 mRNA expression levels upon SMYD3 knockdown in TK10 and 786‐O cells (*n* = 3). *p < 0.05, **p < 0.01, ***p < 0.001, Student's t test. B) Changes in SREBP1 and CD47 mRNA expression levels upon overexpressiong SMYD3 or methyltransferase inactive mutant of SMYD3 (mSMYD3) in TK10 and 786‐O cells. as determined by qPCR (*n* = 3). **p < 0.01, ***p < 0.001, one‐way ANOVA test. C) SREBP1 and CD47 protein expression level changes upon SMYD3 knockdown and overexpression in TK10 and 786‐O cells. D) SREBP1 promoter luciferase activity analysis upon SMYD3 knockdown and overexpression in TK10 and 786‐O cells (*n* = 3). *p < 0.05, **p < 0.01, ***p < 0.001, one‐way ANOVA test. E) Luciferase activity analysis of wild‐type (WT) or SMYD3 motif mutants (MT1–MT5) regulating the SREBP1 promoter in ccRCC cells (*n* = 3). n.s., non‐significant, *p < 0.05, **p < 0.01, Student's t test. F) Quantitative ChIP assay for H3‐K4 tri‐/di‐/monomethylation and Sp1 and SMYD3 occupancy at site 4 of the SREBP1 promoter in ccRCC cells expressing sh‐SMYD3 or control shRNA (*n* = 3). n.s., non‐significant, *p < 0.05, **p < 0.01, ***p < 0.001, Student's *t‐*test. G) Quantitative ChIP assay for H3‐K4 tri‐/di‐/monomethylation and Sp1 and SMYD3 occupancy at site 5 of the SREBP1 promoter in ccRCC cells expressing sh‐SMYD3 or control shRNA (*n* = 3). n.s., non‐significant, *p < 0.05, **p < 0.01, ***p < 0.001, Student's t test. H) Quantitative ChIP assay for H3‐K4 tri‐/di‐/monomethylation and Sp1 and SMYD3 occupancy at site 4 of the SREBP1 promoter in ccRCC cells upon expression of SMYD3, mSMYD3 or control (*n* = 3). n.s., non‐significant, *p < 0.05, **p < 0.01, ***p < 0.001, one‐way ANOVA test. I) Quantitative ChIP assay for H3‐K4 tri‐/di‐/monomethylation and Sp1 and SMYD3 occupancy at site 5 of the SREBP1 promoter in ccRCC cells upon expression of SMYD3, mSMYD3 or control (*n* = 3). n.s., non‐significant, *p < 0.05, **p < 0.01, ***p < 0.001, one‐way ANOVA test. J) CD47 protein expression changes upon SREBP1 knockdown and overexpression in TK10 and 786‐O cells. K) CD47 expression on the cell surface of ccRCC cells stably expressing SREBP1 shRNA, cDNA, or control was determined by flow cytometry. L) CD47 promoter luciferase activity analysis upon SREBP1 knockdown and overexpression in TK10 and 786‐O cells (*n* = 3). *p < 0.05, **p < 0.01, ***p < 0.001, Student's *t*‐test. M) Luciferase activity analysis of wild‐type (WT) or SREBP1 motif mutants (MT1 and MT2) regulating the CD47 promoter in ccRCC cells (*n* = 3). **p < 0.01, ***p < 0.001, Student's *t*‐test. N) Quantitative ChIP assay for SREBP1 occupancy at the SREs of CD47 promoter in RCC cells (*n* = 3). **p < 0.01, ***p < 0.001, Student's *t*‐test. Data are presented as mean ± SEM.

Previous RNA‐sequencing data indicated that SMYD3 could positively regulate the expression of SREBP1. To further verify the regulatory relationship between SMYD3 and SREBP1, using RT‐qPCR and Western blot assays, we measured the expression of SREBP1 in the context of SMYD3‐siRNA mediated knockdown and SMYD3‐cDNA plasmid‐mediated overexpression. As shown in **Figure** **6**A–C, SREBP1 expression was downregulated upon SMYD3 knockdown but upregulated upon SMYD3 overexpression in ccRCC cells.

SMYD3 regulates downstream gene expression at the transcription level. To test whether SMYD3 regulates SREBP1 in this manner, we constructed a SREBP1 promoter luciferase reporter plasmid system (SREBP1‐promoter‐LUC) to perform luciferase reporter assays. We co‐transfected the SREBP1‐promoter‐LUC plasmid with the SMYD3‐knockdown vector or SMYD3‐overexpression vector. As shown in Figure 6D, SMYD3 knockdown markedly suppressed the activation of the SREBP1 promoter, whereas SMYD3 overexpression increased SREBP1 promoter activation. SMYD3 recognizes and binds to the 5′‐CCCTCC‐3′ motif in the promoters of its target genes,^[^ ^14^ ^]^ and we identified five putative SMYD3 binding elements upstream of the transcription start site of the SREBP1 gene (Figure S7C, Supporting Information). Thus, we constructed five luciferase reporter plasmids with a mutant motif (MT1‐MT5) to determine which motif acts as the functional binding element in the promoter of SREBP1. We found that MT4 and MT5 acted as functional binding elements because the MT4 and MT5 mutants abolished the effect of SMYD3 on SREBP1 transactivation in TK10 and 786‐O cells (Figure 6E; Figure S7D, Supporting Information).

### 2.9 SMYD3 Promotes SREBP1 Expression and Sp1 Accumulation in the SREBP1 Promoter via its Histone Methyltransferase‐Dependent Activity

Because SMYD3 promotes target gene transcription by di‐/trimethylating H3‐K4 in the promoter region, we proceeded to determine whether SMYD3 contributed to SREBP1 expression by altering histone modification and subsequent transcription factor recruitment. We then performed a chromatin immunoprecipitation (ChIP) assay to evaluate the status of H3‐K4 methylation in ccRCC cells transfected with SMYD3‐specific shRNA or control shRNA. As shown in Figure 6F,G, SMYD3 knockdown decreased H3‐K4 di‐/trimethylation in the promoter of SREBP1. Our previous studies revealed that SMYD3 recruits the transcription factor Sp1 to the promoters of target genes to synergistically activate their transcription.^[^ ^14b^ ^]^ Moreover, Sp1 is a transcription factor of SREBP1 that promotes SREBP1 transcription by directly interacting with its promoter.^[^ ^27^ ^]^ We therefore evaluated whether SMYD3 knockdown affects the occupancy of Sp1 at the promoter of SREBP1. As expected, SMYD3 knockdown decreased the recruitment of Sp1 to the promoter of SREBP1 (Figure 6F,G). To further prove whether SMYD3 promotes SREBP1 transcription via its histone methyltransferase activity, we conducted a series of experiments with methyltransferase inactive mutant of SMYD3 (mSMYD3), and the data suggested that mSMYD3 could attenuate the expression of SREBP1 caused by wild‐type SMYD3 overexpression, as well as H3K3 di/trimethylation or SP1 recruitment in the SREBP1 promoter region (Figure 6B–D,H,I). Collectively, these results indicate that SMYD3 modifies H3‐K4 di‐/trimethylation and recruits Sp1 to the promoter of SREBP1 to synergistically activate SREBP1 transcription.

### 2.10 CD47 is a Direct Transcriptional Target of SREBP1

Since SREBP1 plays an important role in the transcriptional response of SMYD3, which is involved in mediating its immunosuppressive function, CD47 could be regulated by SREBP1 in ccRCC (Figures 5F and 6J,K). Therefore, we next sought to elucidate the underlying mechanism by which SREBP1 regulates CD47. First, we performed luciferase reporter assays with the CD47 promoter luciferase reporter plasmid system (CD47‐promoter‐LUC) to explore whether SREBP1 could directly regulate the promoter activity of CD47. We co‐transfected a CD47‐promoter‐LUC plasmid with a SREBP1‐knockdown vector or SREBP1‐overexpression vector. As shown in Figure 6L, the promoter activity of CD47 was significantly inhibited upon SREBP1 knockdown and increased upon SREBP1 overexpression in ccRCC cells, indicating that SREBP1 could directly recognize and bind sterol regulatory elements (SREs) in the promoter of CD47. It has been reported that the sequences 5′‐ ATCACCCCAC‐3′ and 5′‐ CTCACACGAG‐3′ in the promoters of target genes could serve as SREs, and these sequences are consistent with the motifs of SREBP1 provided by the JASPAR database (Figure S7E, Supporting Information).^[^ ^28^ ^]^ We then identified two putative SRE sites upstream of the transcription start site of CD47 by using the JASPAR database (Figure S7F, Supporting Information). We subsequently introduced site‐directed mutations into these two SREs in the promoter of CD47 (MT1 and MT2). The promoter activity of CD47 was markedly decreased by either mutant SRE (MT1 or MT2) in transfected ccRCC cells (Figure 6M). Thus, the results of the luciferase reporter assays demonstrated that each of these two sites functions as an SRE. Moreover, we performed ChIP‐qPCR assays with two primers spanning site 1 or site 2 to evaluate which site acts as the functional SRE in the promoter of CD47. As shown in Figure 6N, both sites were occupied by SREBP1, which is consistent with the results of the luciferase reporter assays. Taken together, these results indicate that SREBP1 directly binds to the CD47 promoter to activate gene transcription.

### 2.11 SMYD3 Regulates The Expression of CD47 through SREBP1 to Promote Immune Evasion in ccRCC

**Figure 7:**
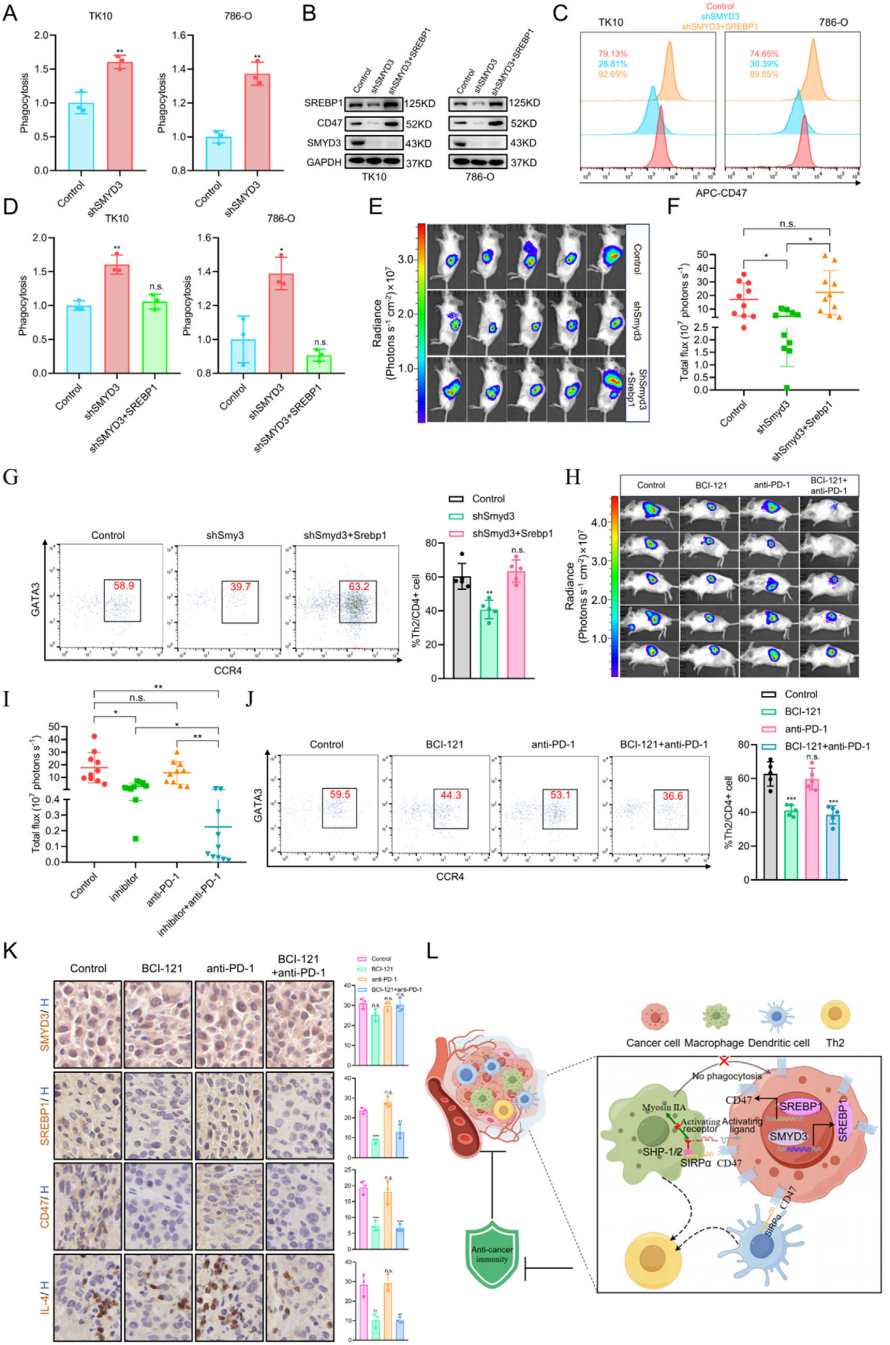
SMYD3 protects renal cancer cells from phagocytosis by monocyte‐derived macrophages, and pharmacological inhibition of SMYD3 enhances the anti‐PD‐1 response: A) The indicated TK10 and 786‐O cells were labeled with the fluorescent dye CFSE, incubated with human peripheral blood monocyte‐derived macrophages for 2 h, and stained with CD11b and analyzed by flow cytometry. The phagocytosis rate was calculated as the percentage of CFSE^+^CD11b^+^ cells in CSFE^+^ cells (*n* = 3), and the phagocytosis rate in control cells was set as 100%. **p < 0.01, Student's *t*‐test. B) The inhibitory effect on CD47 expression caused by SMYD3 knockdown could be reversed by SREBP1 overexpression in ccRCC cells. C) The decrease in CD47 expression on the cell surface caused by SMYD3 knockdown was abolished by SREBP1 overexpression in ccRCC cells. D) The indicated TK10 and 786‐O cells were subjected to a phagocytosis assay similar to that described in A (*n* = 3). n.s., non‐significant, *p < 0.05, **p < 0.01, one‐way ANOVA test. E) Representative bioluminescence images of tumors in BALB/c mice orthotopically engrafted with control, SMYD3‐knockdown, and rescued SREBP1‐overexpressing RENCA tumors. F) Burden of control, SMYD3‐knockdown, and rescued SREBP1‐overexpressing RENCA tumors as measured by bioluminescence (*n* = 10). n.s., non‐significant, *p < 0.05, one‐way ANOVA test. G) Flow cytometry gating and frequency of Th2 cells among total CD4^+^ T cells in RENCA tumors in the indicated groups (*n* = 5). n.s., non‐significant, **p < 0.01, one‐way ANOVA test. H) Representative bioluminescence image of Day 16 tumors in mice with RENCA tumors treated with vehicle, the SMYD3 inhibitor BCI‐121 (1 mg kg^−1^, i.p., daily), anti‐PD‐1 mAb (10 mg kg^−1^, i.p., on Days 1, 4, 7 and 14) or a combination of BCI‐121 and anti‐PD‐1 mAb. I) Burden of RENCA tumors treated with vehicle, BCI‐121, an anti‐PD‐1 mAb, or a combination of both (*n* = 10). n.s., non‐significant, *p < 0.05, **p < 0.01, one‐way ANOVA test. J) Flow cytometry gating and frequency of Th2 cells among total CD4^+^ T cells in RENCA tumors treated with either vehicle, BCI‐121, anti‐PD‐1 mAb, or a combination of BCI‐121 and anti‐PD‐1 mAb (*n* = 5). n.s., non‐significant, ***p < 0.001, one‐way ANOVA test. K) Representative images of orthotopic syngeneic mouse tumors stained for SMYD3, SREBP1, CD47, and IL‐4 and quantification of IHC staining (*n* = 3). n.s., non‐significant, **p < 0.01, ***p < 0.001, one‐way ANOVA test. L) Proposed schematic representation depicting the mechanism by which SMYD3 transcriptionally activates SREBP1‐CD47 to promote Th2 cell infiltration‐induced immune evasion in ccRCC. Data are presented as mean ± SEM.

SREBP1 is the downstream transcription factor of SMYD3 that regulates CD47 expression in ccRCC, and CD47 is well characterized as an inhibitory checkpoint target expressed on the surface of cancer cells. CD47 is an important anti‐phagocytosis regulator of macrophages in the innate immune system.^[^ ^29^ ^]^ Thus, we sought to explore the role of SMYD3 in protecting tumor cells against clearance by the immune system in ccRCC. The indicated CFSE‐labeled TK10 and 786‐O cells were cocultured with human peripheral blood monocyte‐derived macrophages and analyzed by FACS. SMYD3 knockdown significantly increased phagocytosis in TK10 and 786‐O cells (**Figure** **7**A). To determine whether SMYD3 drives tumor immune evasion via CD47 upregulation and subsequent activation of SREBP1 expression, we overexpressed SREBP1 in stable SMYD3‐knockdown 786‐O and TK10 cells to investigate whether the effects of SMYD3 knockdown could be eliminated upon SREBP1 overexpression (Figure 7B,C). The clearance of tumor cells by phagocytes caused by SMYD3 knockdown was abolished by SREBP1 overexpression in ccRCC (Figure 7D). Additionally, the tumorigenicity of shSmyd3 cells increased upon Srebp1 overexpression and was comparable to that of nontarget controls in an orthotopic syngeneic mouse tumor model in vivo (Figure 7E,F), and the pattern of Th2 cell infiltration was similar to the tumorigenicity (Figure 7G; Figure S8, Supporting Information).

### 2.12 Pharmacological Inhibition of SMYD3 Strengthens the Anti‐PD‐1 Response in ccRCC

Since upregulated SMYD3 shapes the immunosuppressive microenvironment in ccRCC through the SREBP1‐CD47 axis, pharmacological inhibition of SMYD3 may have a synergistic effect with PD‐1 inhibitors to achieve increased tumor therapeutic efficacy. BCI‐121, a specific inhibitor of SMYD3, can reduce the di‐ and trimethylation of H3K4 mediated by SMYD3, thereby downregulating SMYD3 target gene transcription.^[^ ^30^ ^]^ BCI‐121 could inhibit SREBP1 and CD47 expression, as well as promote phagocytosis in TK10 and 786‐O cells (Figure S9, Supporting Information).To determine whether BCI‐121 can restore sensitivity to PD‐1 inhibitor therapy, we intraperitoneally (i.p.) administered BCI‐121 and an anti‐PD‐1 antibody to RENCA orthotopic syngeneic model mice. We used the IVIS lumina system to evaluate the effect of combination therapy with BCI‐121 and anti‐PD‐1 antibodies in vivo, and combination therapy further reduced tumor size, indicating that BCI‐121, which inhibits SMYD3 activity, can sensitize tumors to anti‐PD‐1 therapy, thus producing a synergistic effect (Figure 7H,I). We also found that BCI‐121 downregulated the expression of SREBP1 and CD47 and reduced the infiltration of Th2 cells, thereby inhibiting the secretion of IL‐4 (Figure 7J,K).

Overall, we delineated the role of the cancer cell‐intrinsic SMYD3‐SREBP1‐CD47 axis in promoting renal cancer cell escape from immune attack (Figure 7L). SMYD3 modifies H3‐K4 di‐/trimethylation and recruits Sp1 to the promoter of SREBP1 to synergistically activate SREBP1 transcription. As a TF, SREBP1 recognizes and binds two SREs in the promoter of CD47 to promote gene transcription, and CD47 acts as an inhibitory checkpoint to mediate immune evasion in ccRCC.

## 3 Discussion

ICIs and ICI‐based combination therapies have significantly improved clinical outcomes in patients with ccRCC.^[^ ^6^ ^]^ However, this benefit is limited to small subsets of ccRCC patients, most of whom acquire drug resistance even if they have high levels of immune checkpoint molecule expression (e.g., PD‐L1).^[^ ^31^ ^]^ This situation poses considerable challenges for ccRCC immunotherapy. The efficacy of ICIs in patients with ccRCC may depend on two factors.^[^ ^16^, ^32^ ^]^ On the one hand, ccRCC patients express immune checkpoint molecules as therapeutic targets; on the other hand, the antitumor immune microenvironment, such as increased infiltration of activated CD8+ T cells and Th17 cells and decreased infiltration of Th2 cells in the immune microenvironment of ccRCC, is favorable.^[^ ^4^ ^]^ For ccRCC patients with high expression of immune checkpoint molecules according to a pan‐cancer analysis,^[^ ^4^, ^33^ ^]^ it is particularly important to have a microenvironment that promotes antitumor immunity rather than immunosuppression. In this study, we revealed that cancer cell‐intrinsic SMYD3 impaired the response of ccRCC to a PD‐1 inhibitor by affecting the infiltration of immune cells in the tumor microenvironment shaped by immunosuppression.

Th2 cells play an immunosuppressive role in the TME and contribute to tumor progression by recruiting and activating immunosuppressive cells. For example, Th2 cells infiltrate the pancreas in the early stages of tumorigenesis and secrete IL‐4 and IL‐13, which bind to IL‐4R and IL‐13R to activate JAK1‐STAT6 pathway to increase glycolysis of cancer cells to fuel tumor progression in a murine Kras^G12D^‐driven PDAC model.^[^ ^21a^ ^]^ A Th2‐associated fibrotic TME was found in low tumor‐infiltrating lymphocytes (TILs) with an increased Th2/Th1 ratio, upregulated fibrosis growth factors, and stromal enrichment of cancer‐associated fibroblasts, which indicates that high Th2 cells reduce immune infiltration into tumors and inhibit immune checkpoint blockade (ICB) efficacy.^[^ ^34^ ^]^ Moreover, pharmaceutical inhibition of Th2 cell function could promote the ICB response by remodeling the immune landscape of the TME.^[^ ^35^ ^]^ Additionally, Th2 cells secrete IL‐4 and IL‐10 to induce M2 polarization of macrophages, which increases tumor progression and IL‐4 secretion, thereby establishing a positive feedback loop.^[^ ^36^ ^]^ Besides, Th2 and Treg cell levels generally showed a positive correlation with mutation load. These correlations could be indicative of an immunosuppressive environment enriched in Treg and/or Th2 cells where tumors have escaped elimination by the immune system despite bearing a large number of potentially immunogenic mutations. This indicates Th2 cells play an immunosuppressive role independent of mutation load in ccRCC.^[^ ^4^ ^]^ In summary, our results have demonstrated that Th2 CD4^+^ T cells could play an immunosuppressive role in regulating the TME of ccRCC.

In our previous studies, SMYD3, a vital onco‐driver, was found to play crucial roles in tumorigenesis and progression in prostate cancer as well as RCC.^[^ ^14^ ^]^ However, the effects of SMYD3 on the tumor immune microenvironment and the response of ccRCC to ICIs have not been investigated. In this study, we found that upregulated SMYD3 expression was closely related to the tumor immune microenvironment, orchestrating an inhibitory immune microenvironment in ccRCC by affecting the transcriptome profiles of tumor‐infiltrating immune cells and the landscape of immune cell infiltration (e.g., Th2 cells) in the TME of ccRCC. Recently, we noticed that the immunomodulatory role of SMYD3 has been reported in the HPV‐negative head and neck squamous cell carcinoma mouse models.^[^ ^17^ ^]^ In their study, researchers found that while Smyd3 anti‐sense oligonucleotides (ASOs) did not affect the cell abundance of tumor infiltrating immune cells, they did influence the functional status of these cells by impacting their transcriptional profiles. Smyd3 ASOs reinvigorated CD8+ T‐cells and promoted anti‐tumor neutrophils, as well as a Treg and M2 macrophage phenotype in vivo. Our study revealed that SMYD3 not only alters the transcriptional profile of immune cells, such as influencing the expression of cebpb in neutrophils and macrophages to exert an immunosuppressive function, but also impacts Th2 cell infiltration in TME. The differing immunomodulatory effects of SMYD3 observed in these two studies can be attributed to several factors. First, the tumor models utilized were different, Tsai's study focused on HPV‐negative head and neck squamous cell carcinoma, while our study examined a kidney cancer model. This highlights the heterogeneous nature of the immune microenvironment across various tumor types. Secondly, our research specifically investigated the impact of cancer cell‐intrinsic SMYD3 on the tumor immune microenvironment. We achieved this by constructing a tumor cell line with stable SMYD3 knockdown, without altering the expression levels of SMYD3 in the immune cells. Tsai's study aimed to observe the immunomodulatory function of SMYD3 by administering anti‐sense oligonucleotides treatment in mice, which impacted the expression of SMYD3 in both tumor and immune cells.

Mechanistically, SMYD3 modified H3‐K4 di‐/trimethylation and recruited Sp1 to promote the transcription of SREBP1, which further promoted immune evasion by directly regulating the expression of CD47. CD47 is an innate immune checkpoint ligand that is expressed in many types of cancers, and plays a crucial role in macrophage/dendritic cell‐mediated phagocytosis upon binding to the receptor SIRPα, which is expressed mainly on myeloid cells.^[^ ^24^, ^37^ ^]^ Moreover, CD47 can influence the infiltration of immune effector cells (e.g., T cells) in the TME because of its role as a bridge between innate and adaptive immunity.^[^ ^24b^ ^]^ Our results suggest that CD47, an inhibitory immune molecule downstream of SMYD3, mediates the immunosuppressive function of SMYD3 in ccRCC. Although we confirmed that CD47 expressed on the tumor cell surface can promote Th2 cell infiltration in the TME, the underlying mechanism of CD47 promoting the Th2 cell‐mediated suppression of anti‐tumor immunity is not defined. The main role of CD47, as a bridge between innate and adaptive immunity, occurs in its interaction with antigen presenting cells (e.g., dendritic cells). Therefore, it is reasonable to hypothesize that dendritic cells play an important role in CD47‐mediated Th2 cell infiltration, which needs to be explored in the future.

SREBP1, encoded by SREBP1, is a transcription factor that promotes immunosuppressive (M2‐like) tumor‐associated macrophage (TAM) survival and tumor immune evasion by mediating fatty acid synthesis in M2 macrophages.^[^ ^38^ ^]^ Furthermore, SREBP1 blockade was found to increase the efficacy of immune checkpoint inhibitors.^[^ ^38^ ^]^ In this study, we focused on SREBP1 in ccRCC cells to investigate its function in the tumor immune microenvironment. We found that SREBP1 could recognize and directly bind to two SREs in the promoter of CD47 to activate CD47 transcription. The upregulation of CD47 expression by SREBP1 inhibited the phagocytosis of tumor cells, resulting in a reduction in the infiltration of immune effector cells, which in turn promoted tumor immune evasion in ccRCC. Therefore, we propose that the combined inhibition of SREBP1 and an immune checkpoint might achieve greater anti‐tumor efficacy in ccRCC.

CD47, an innate inhibitory checkpoint molecule, enables tumor cells to evade immune surveillance.^[^ ^24a^ ^]^ To date, the results of several clinical trials have suggested that anti‐CD47 therapy is a promising clinical strategy for patients with hematological malignancies, with a high ORR.^[^ ^39^ ^]^ Furthermore, a preclinical study revealed that the combination of a PD‐1 inhibitor with anti‐CD47 therapy could maximize antitumor efficiency in solid tumors.^[^ ^40^ ^]^ However, the clinical application of anti‐CD47 therapy is limited by its side effects, especially its toxic effect on red blood cells (RBCs), because CD47 is widely expressed on the surface of RBCs.^[^ ^24a^ ^]^ Moreover, this limitation is particularly pronounced in solid tumors because of the antigen sink effect. The widespread expression of CD47 in normal tissue means that a drug may need to be administered as a large initial dose and/or frequently to effectively block CD47 activity in cancer cells.^[^ ^39^ ^]^ To overcome these limitations, the upstream regulators of CD47 in tumor cells need to be elucidated, and alternative treatments targeting these regulators may achieve a balance between antitumor efficacy and toxicity. We found SMYD3 is an upstream regulator of CD47 and SMYD3 does not bind to the promoter region of CD47 directly, indicating that SMYD3 regulates CD47 expression through epigenetic regulators that affect gene transcription, including chromatin modifiers (methyltransferases, acetyltransferases, ubiquitinases et al. and their erasing enzymes), DNA methyltransferase and chromatin remodelers et al. Our findings confirmed that SMYD3 can affect the transcription of CD47 by directly regulating the expression of SREBP1. However, whether SMYD3 regulates CD47 through other regulators except transcription factors needs to be further investigated. More importantly, the expression of SMYD3 is undetectable or very weak in most types of normal human tissue and significantly elevated in tumor tissue (e.g., colorectal cancer, hepatocellular carcinoma, prostate cancer, breast cancer, and ccRCC),^[^ ^14^, ^41^ ^]^ which makes SMYD3 an ideal target with relatively few off‐target effects. As mentioned above, dual checkpoint blockade targeting CD47 and PD‐L1 has a synergistic effect because it activates both innate immunity and adaptive immunity to achieve antitumor effects. Similarly, our preclinical model suggests that the alternative approach of targeting SMYD3 to affect CD47 signaling combined with a PD‐1 inhibitor results in increased tumor suppression in ccRCC.

In summary, in addition to affecting the proliferation of tumor cells themselves, cancer cell‐intrinsic SMYD3 shapes the immunosuppressive microenvironment by altering the landscape of immune cell infiltration, thus impairing the efficacy of ICI therapy (PD‐1 blockade). Moreover, we identified CD47, an innate immune checkpoint molecule expressed on tumor cells, as the downstream molecule of SMYD3 that inhibits anti‐tumor immunity in ccRCC. Thus, combined therapy to block both SMYD3 and PD‐1 could be a promising regimen for reducing the off‐target effects caused by anti‐CD47 therapy.

## 4 Experimental Section

### Patients and Specimens

A cohort of 30 patients with ccRCC were included who had undergone radical nephrectomy at Qilu Hospital of Shandong University. Informed consent was obtained from all participants. Renal tumors and matched normal tissues were collected under experimental protocols approved by the Ethics Committee on Scientific Research of Shandong University, Qilu Hospital. The corresponding clinical and pathological characteristics are summarized in Table S4 (Supporting Information). Additionally, a TMA containing 90 ccRCC tissues and matched normal tissues with follow‐up data was obtained from Shanghai Outdo Biotech (Shanghai, China). The corresponding clinical and pathological characteristics are summarized in Table S5 (Supporting Information).

### Cell Lines

786‐O cells derived from the primary tumor of a 58‐year‐old white male patient with renal adenocarcinoma were purchased from the Cell Bank of the Chinese Academy of Sciences (Shanghai, China), TK10 cells derived from the primary tumor of a 43‐year‐old male patient with renal cell adenocarcinoma were purchased from Procell (Wuhan, China), and RENCA cells isolated from the kidney of a male BALB/c mouse with spontaneous renal cortical adenocarcinoma were obtained from the American Type Culture Collection (ATCC; Manassas, VA, USA). All the cell lines were cultured in RPMI 1640 medium (11 875 093, Gibco, Grand Island, NY, USA) supplemented with 10% FBS (10099‐141, Gibco), 100 U mL^−1^ penicillin (V900929, Sigma‐Aldrich, St. Louis, MO, USA), and 0.1 mg mL^−1^ streptomycin (V900929, Sigma‐Aldrich) at 37 °C in 5% CO_2_. The culture medium for RENCA cells also contained 1× GlutaMAX (35 050 061, Gibco), 1 mmol L^−1^ sodium pyruvate (11 360 070, Gibco) and 1× nonessential amino acids (11 140 050, Gibco). The cell lines were routinely confirmed to be free of mycoplasma and authenticated by STR detection.

### Animals

Five‐ to six‐week‐old male BALB/c mice were purchased from Charles River Laboratories (Beijing, China). The experimental animals were housed under specific pathogen free (SPF) conditions with food and water ad libitum. Animal experiments were performed in strict accordance with the Guidelines of Ethics Committee on Scientific Research of Shandong University, Qilu Hospital.

### Small‐Interfering RNA (siRNA) and Plasmid Transfection

Transfection assays were performed as previously described.^[^ ^14^ ^]^ For siRNA transfection, cells were seeded in 6‐well plates and transfected with siRNA (GenePharma, Shanghai, China) or control siRNA using jetPRIME (101 000 046, Polyplus, Illkirch, France). To overexpress target genes in RCC cells, the pcDNA3.1 vector containing target gene cDNAs (GeneChem, Shanghai, China) was transiently transfected into the cells via jetPRIME. The methyltransferase inactive of SMYD3 mutants was introduced via Site‐Directed Mutagenesis Kits (Thermo Fisher Scientific). All siRNA sequences are presented in Table S6 (Supporting Information).

### Lentiviral Infection

To construct stable Smyd3‐ and Cd47‐knockdown RENCA cells, stable Srebp1‐ and Cd47‐overexpressing RENCA cells, stable SREBP1‐overexpressing TK10 and 786‐O cells, and stable SMYD3‐, SREBP1‐knockdown TK10 and 786‐O cells were infected with lentivirus as indicated. Lentiviral vectors containing Smyd3/Cd47 mouse shRNA, SMYD3/SREBP1 human shRNA, Srebp1/Cd47 mouse cDNA, and SREBP1 human cDNA were purchased from WZ Biosciences (Jinan, China). All shRNA sequences are presented in Table S6 (Supporting Information).

### Real‐Time Quantitative Polymerase Chain Reaction (RT‐qPCR)

RNA was extracted using TRIzol according to the manufacturer's instructions (T9108, TaKaRa, Dalian, China). One microgram of total RNA was used for cDNA synthesis by the PrimeScript RT reagent Kit with gDNA Eraser (RR047A, TaKaRa). qPCR was performed with TB Green Premix Ex Taq II (RR820B, TaKaRa) on a LightCycler 960 system (Roche, Mannheim, Germany). The mRNA level of each target gene was analyzed on the basis of the corresponding CT values and normalization to β‐Actin expression. The primers used in this study are shown in Table S7 (Supporting Information).

### Western Blot

Western blot was performed as previously described.^[^ ^14^ ^]^ Briefly, equal amounts of proteins were loaded onto an SDS‐PAGE gel. The separated proteins were transferred to a PVDF membrane (Millipore, MA, USA). After being blocked with 5% BSA in TBS, the membrane was incubated with a primary antibody at 4 °C overnight. The membrane was subsequently exposed to horseradish peroxidase‐labeled secondary antibodies (1:5000) for 1 h at room temperature and imaged with western ECL Substrate (Millipore) via a chemiluminescence detection system (Amersham, Pittsburgh, PA). The following primary antibodies were used in this study: anti‐SMYD3(ab187149, Abcam, Cambridge, MA, USA), anti‐SREBP1(14088‐1‐AP, Proteintech, Wuhan, China), anti‐CD47(NBP2‐31106, Novus, Littleton, CO, USA), and anti‐GAPDH (10494‐1‐AP, Proteintech). HRP‐conjugated AffiniPure Goat anti‐rabbit IgG(H+L) (SA00001‐2, Proteintech) was used as the secondary antibody.

### Single‐cell RNA Sequencing (scRNA‐seq)

scRNA‐seq was performed on fluorescence‐activated cell sorting (FACS)‐enriched live CD45+ cells from tumors of orthotopic RENCA‐bearing mice. To generate single‐cell libraries, a single‐cell suspension was added to the 10X Genomics Chromium Single Cell 3 Kit (v3), with the expectation of capturing 8000 cells. cDNA amplification and library construction were performed according to standard protocols. Libraries were sequenced by LC‐Bio Technology (Hangzhou, China) on an Illumina NovaSeq 6000 sequencing system (double‐end sequencing, 150 bp) at a minimum depth of 20000 reads per cell.

The results from Illumina sequencing offline were converted to FASTQ format using bcl2fastq software (version 5.0.1). CellRanger software was used to compare the FASTQ data with the reference genome, and the 3′ end transcripts of individual cells were identified and counted in the sequenced samples. (https://support.10xgenomics.com/single‐cell‐geneexpression/software/pipelines/latest/what‐is cell‐ranger, version 7.0.0). The output CellRanger expression profile matrix was loaded into Seurat (version 4.1.0) to filter low‐quality cells from the scRNA‐seq data, and the filtered data were downscaled and clustered. Filtering low‐quality thresholds were as follows: number of genes expressed per cell >500 and mitochondrial genes expressed in <25% of cells. The singleR database and scCATCH database were used to identify each cluster cell type. Further resolution of the cell types was carried out via the use of known gene markers associated with different immune cell subtypes.

### RNA Sequencing

RNA was extracted from SMYD3‐knockdown and control TK10 cells. The RNA quantification, qualification, cDNA library preparation, and subsequent RNA sequencing were conducted by LC‐BIO Co., Ltd. (Hangzhou, China) according to the standard Illumina RNA‐seq protocol. FeatureCounts v1.5.0‐p3 was used to count the read numbers mapped to each gene. A fold change > 1.5 and an FDR < 0.05 were set as the thresholds for differentially expressed gene (DEG) analysis.

### Dual‐Luciferase Reporter Assay

Cells were seeded in 6‐well plates at 2 × 10^5^ cells per well and transfected with a reporter plasmid (pGL3‐SREBP1‐Luc reporter plasmid and pGL3‐CD47‐Luc reporter plasmid) and the pRL‐TK plasmid with or without SMYD3/SREBP1 siRNA or overexpression plasmids. After 48 h of incubation, luciferase activity was measured via a dual‐luciferase reporter assay system following the manufacturer's instructions (Promega, Madison, WI), and the signal was normalized to that of the Renilla luciferase control as relative luciferase units. All reporter plasmids used in this study were purchased from Biosune Biotechnology Co., Ltd (Shanghai, China). Putative binding sites in the promoter of each target gene were identified via the TF database JASPAR (https://jaspar.genereg.net/), and mutant variants were made using Site‐Directed Mutagenesis Kits (Thermo Fisher Scientific).

### Data Source and Handling

The RNA sequencing data and corresponding clinical information of ccRCC patients were downloaded from the TCGA database (http://portal.gdc.cancer.gov/). Other gene expression profile data were obtained from the Gene Expression Omnibus (GEO) (http://www.ncbi.nlm.nih.gov/geo), Oncomine (http://www.oncomine.org/resource/login.html), UALCAN (http://ualcan.path.uab.edu/analysis.html) and CPTAC (http://proteomics.cancer.gov/programs/cptac) databases. The E‐MTAB‐1980 cohort included 101 patients with ccRCC, and RNA array data and clinical information were downloaded from http://www.ebi.ac.uk. The ICGC‐RECA‐EU cohort, which included 91 patients with ccRCC, was downloaded from http://dcc.icgc.org/. One ccRCC immunotherapy dataset was further selected, IMmotion151.^[^ ^22^ ^]^ IMmotion151 contains data from 823 patients with ccRCC who received ICIs plus bevacizumab, and sunitinib, and data from a subgroup of 407 patients who received atezolizumab in combination with bevacizumab were used for further analysis in this study. Patients with complete/partial remission (CR or PR, respectively) were divided into the response group, and patients with progressive disease (PD) were divided into the non‐response group. The IMmotion151 dataset was downloaded from the European Genome‐phenome Archive (EGA) (http://ega‐archive.org/), as allowed. The relationships between gene expression and survival (disease‐free survival and overall survival) in RCC patients were analyzed via the online database KM plotter (http://kmplot.com/analysis/index.php?p=service). The Survminer package was used to determine the best cutoff for survival analysis (Log‐rank test) in the IMmotion151 cohort, and this cutoff was used for subsequent immune cell infiltration evaluation and IL‐4/IL‐13 score analysis in the IMmotion151 cohort.

### Immune Infiltration Evaluation

ssGSEA was employed to calculate the abundance of immune cells. For the algorithm of ssGSEA, immune cell gene sets were downloaded from the study of Charoentong.^[^ ^42^ ^]^ The abundance of immune cells was represented by enrichment scores.

### Chromatin Immunoprecipitation (ChIP) Assay

ChIP assays were performed with a ChIP Assay Kit (17‐10086, Millipore, Billerica, MA, USA) according to the manufacturer's protocol. In brief, pretransfected cells were crosslinked with 1% formaldehyde, and the reaction was terminated by the addition of glycine. The cells were subsequently harvested with SDS lysis buffer, and their chromatin was fragmented via ultrasonic shearing. Then, the chromatin was separated by agarose gel electrophoresis to generate DNA fragments with an average size of 200–1000 bp. The samples were incubated with a mixture of an antibody and protein‐A/G beads at 4 °C overnight. Washing and crosslinking reversal steps were performed, followed by DNA extraction, precipitation, and analysis by RT‐qPCR. The antibodies used for ChIP analysis recognized the following targets: SMYD3 (sc‐398085, Santa Cruz Biotechnology, Santa Cruz, CA, USA), SREBP1 (14088‐1‐AP, Proteintech), H3‐K4 Me1 (#5326, Cell Signaling Technology, Danvers, MA, USA), H3‐K4 Me2 (#9725, Cell Signaling Technology), H3‐K4 Me3 (#9751, Cell Signaling Technology), and Sp1 (#9389, Cell Signaling Technology). The PCR primer sequences are listed in Supplementary Table S7.

### Isolation of Monocyte‐Derived Macrophages

Peripheral blood mononuclear cells (PBMCs) were isolated from healthy donors via Ficoll‐Hypaque (17144002GE Healthcare, Little Chalfont, Buckinghamshire, UK). CD14^+^ monocytes were subsequently isolated by magnetic column purification via anti‐CD14 microbeads (130‐050‐201, Miltenyi Biotec, Bergisch Gladbach, Germany). After that, 1 × 10^6^ CD14^+^ cells were cultured for 7 days in RPMI‐1640 medium supplemented with 10% FBS, 2 mm glutamine, 1% penicillin per streptomycin, and 30 ng mL^−1^ macrophage colony‐stimulating factor (M‐CSF) (416‐ML, R&D Systems, Minneapolis, MN, USA).

### Phagocytosis Assay

Macrophages were plated (5 × 10^4^ per well) in a 24‐well tissue‐culture plate in complete RPMI‐1640 medium, supplemented with M‐CSF 24 h before any experiment. RCC cells were stained with 2.5 µm carboxyfluorescein succinimidyl ester (CFSE) according to the manufacturer's protocol (abs9106, Absin, Shanghai, China). The macrophages were incubated in serum‐free medium for 2 h before 2 × 10^5^ CFSE‐labeled RCC cells were added. After coculture for 2 h at 37 °C, the cells were harvested, the macrophages were stained with an APC‐conjugated anti‐CD11b antibody (561 015, BD, San Jose, CA, USA), and flow cytometry was performed. The phagocytosis rate was calculated as the percentage of CD11b+CFSE^+^ cells among CSFE^+^ cells.

### Flow Cytometry

To isolate single cells from orthotopic syngeneic tumors, tumor tissues were dissociated for 1 h at 37 °C by digestion with LiberaseTM TL (0 540 102 0001, 0.5 mg mL^−1^, Roche), DNase I (10 104 159 001, 10 µg mL^−1^; Roche) and Dispase II (4 942 078 001, 2 mg mL^−1^, Roche) in RPMI medium. Dispersed cells were passed through a 70‐µm strainer and pelleted by centrifugation; then, these cells were resuspended, layered onto a Percoll density gradient (17‐0891‐02, GE Healthcare), and centrifuged for 15 min at 2500 rpm. Mononuclear cells were isolated through the collection of the interphase fraction between 40% and 80% Percoll. The cells were subsequently incubated with Zombie Aqua (423 101, BioLegend, San Diego, CA, USA) to assess cell viability. Next, the cells were incubated with a CD16/CD32 antibody (553 141, BD Biosciences) to block FcgR binding for 10 min and then with the antibody mixture for 30 min at room temperature. Fluorochrome‐conjugated antibodies against CD45 (56‐0454‐81, Thermo), CD3 (100 235, BioLegend), CD4 (100 421, BioLegend), CCR4 (131 203, BioLegend), and GATA3 (653 812, BioLegend) were used. All samples were acquired with Gallios (Beckman, Miami, FL, USA) and analyzed with FlowJo software (TreeStar, Ashland, OR, USA).

### Immunohistochemistry

Immunohistochemistry was performed as previously described.^[^ ^14^ ^]^ After deparaffinization and hydration, the tissue sections were immersed in sodium citrate antigen retrieval solution for antigen retrieval. Blocking of endogenous peroxidase activity and nonspecific antibody binding was carried out, followed by incubation with a primary antibody overnight at 4 °C in a humidified chamber. DAB development was carried out after the slides were incubated with a secondary antibody labeled with HRP. Finally, the slides were counterstained with hematoxylin, dehydrated with ethanol, cleared with xylene, and mounted with resin mounting medium. The antibodies used for IHC recognized the following targets: SMYD3 (ab187149, Abcam), SREBP1 (14088‐1‐AP, Proteintech), CD47 (NBP2‐31106, Novus), and IL‐4 (66142‐1‐Ig, Proteintech).

### Multiplex Immunohistochemistry

To explore the correlation between the protein of interest and Th2 cell infiltration in ccRCC, TMA sections from ccRCC and adjacent normal tissues (details given in Table S5 (Supporting Information)) were subjected to multiplex immunohistochemistry. Briefly, TMA sections were heated at 65 °C for 60 min and subjected to standard dewaxing and rehydration (through a graded series of ethanol solutions: 100% 2 × 5 min; 90% 1 × 5 min; 70% 1 × 5 min; and rinsing in DI water). Heat‐induced antigen retrieval was performed in a microwave oven. For multiplex immunofluorescence staining, TMA sections were stained with panels containing antibodies against CK (ZM‐0069, ZSGB‐BIO, working solution), CD4 (48274S, Cell Signaling Technology, dilution 1:100), GATA3 (ab199428, Abcam, dilution 1:200), CD47 (ab218810, Abcam, dilution 1:500), and SMYD3 (ab187149, Abcam, dilution 1:100). All the markers were stained in sequence using their respective fluorophores contained in the Opal 6‐Plex Detection Kit (NEL821001KT, Akoya Biosciences, MA, USA) according to the manufacturer's protocol. The stained slides were scanned using the Polaris imaging system (Akoya Biosciences). Image analysis was performed in QuPath V.0.4.2 (Queen's University Belfast, Northern Ireland, UK). The cells were phenotyped into different classes by positivity thresholds for each marker set based on cytoplasmic or nuclear staining intensity. The cell count, density, and percentage were calculated for each phenotype (Table S8). GATA3 are well‐recognized markers of Th2 cells, and CD4/GATA3‐positive cells were regarded as Th2 cells.^[^ ^34^ ^]^

### Orthotopic Renal Cell Carcinoma Mouse Model

Animal experiments were performed in strict accordance with the Guidelines of Ethics Committee on Scientific Research of Shandong University, Qilu Hospital. Five‐ to six‐week‐old male BALB/c mice were purchased from Charles River Laboratories. An orthotopic renal cell carcinoma mouse model was generated according to the previous studies.^[^ ^43^ ^]^ The mice were subjected to gaseous anesthesia induced by isoflurane and then fixed in a prone position. The mice were disinfected on the left side of the back and spine, and a 1.5 cm longitudinal incision was made at the costal margin. The surrounding tissues were mobilized, and the kidney was squeezed through the incision. A needle was inserted between the kidney parenchyma and capsule, and a total of 8 × 10^5^ luciferase‐engineered RENCA cells (shSmyd3 and control) were injected. The muscle and skin were sutured layer by layer, followed by routine disinfection. The mice were randomized into 2 groups and treated as follows: (i) vehicle control and (ii) anti‐PD‐1 (10 mg kg^−1^, i.p.). The anti‐PD‐1 antibody (BE0146, clone RMP1‐14, Bio X Cell, West Lebanon, NH, USA) or isotype control antibody (BE0089, clone 2A3, rat IgG2a, Bio X Cell) was i.p. injected on Days 1, 4, 7, and 14. The mice were also weighed 1 to 2 times per week to monitor for signs of drug toxicity. Tumors were measured via bioluminescence imaging. For bioluminescence imaging, the mice were injected intraperitoneally with firefly d‐luciferin at 150 mg kg^−1^ and images were acquired 10 min after luciferin injection using an IVIS Spectrum (Perkin Elmer). Total flux was quantified using Living Image 4.0 software. Other animal experiments to explore the effects of target genes on tumor growth were performed as described above.

### Quantification and Statistical Analysis

Statistical analysis was performed using GraphPad Prism software (GraphPad Software, Inc., San Diego, CA, USA) or R. Data are expressed as the mean ± SEM and were analyzed using unpaired or paired Student's *t*‐test for two‐group, and one‐way ANOVA for multigroup comparisons. The Kaplan‐Meier survival analysis was performed by using the log‐rank test. For categorical variables, the chi‐square test or rank sum test was performed. Coefficients of Spearman's rank correlation or Pearson's correlation were calculated to describe the correlation of two variables. p values and FDR values <0.05 were considered significant.

## Conflict of Interest

The authors declare no conflict of interest.

## Author Contributions

S.C., B.H., B.S., and N.Z. designed and supervised the study; Z.L., X.Z., and M.Z. carried out the majority of experiments; H.Y. and X.Q. performed bioinformatics analysis; X.L. and S.Z. performed orthotopic syngeneic mouse model arrays; R.T., K.Y., L.L., and Y.F. technically supported and revised the manuscript. All authors contributed to the data analysis. Z.L. and X.Z. wrote the manuscript. All authors reviewed and gave final approval of the manuscript.

## Supporting information



Supporting Information

Supplemental Table 1

Supplemental Table 2

Supplemental Table 3

Supplemental Table 4

## Data Availability

The data that support the findings of this study are available from the corresponding author upon reasonable request.
