# Supplementary material for: SMYD3 Promotes Immune Evasion in Clear Cell Renal Cell Carcinoma via SREBP1‐Mediated Transactivation of CD47
Source: Adv Sci (Weinh). 2025 Jun 23;12(34):e04200. doi: 10.1002/advs.202404200 (PMC12442605; doi:10.1002/advs.202404200)
Supplement: Supplementary file 1 — Supporting Information [file ADVS-12-e04200-s004.docx]

SMYD3 promotes immune evasion in clear cell renal cell carcinoma via SREBP1-mediated transactivation of CD47

Zhengfang Liu, Xiumei Zhao, Maolin Zang, Huiyang Yuan, Xin Qin, Xiaofeng Li, Shuo Zhao, Ruirong Tan, Keqiang Yan, Li Liu, Yidong Fan, Ning Zhang^*^, Benkang Shi^*^, Bo Han ^*^ and Shouzhen Chen ^*^

**This PDF file includes Figure S1-S9 and Table S4-S7.**

**Supplementary Figures**

**Figure S1**

**
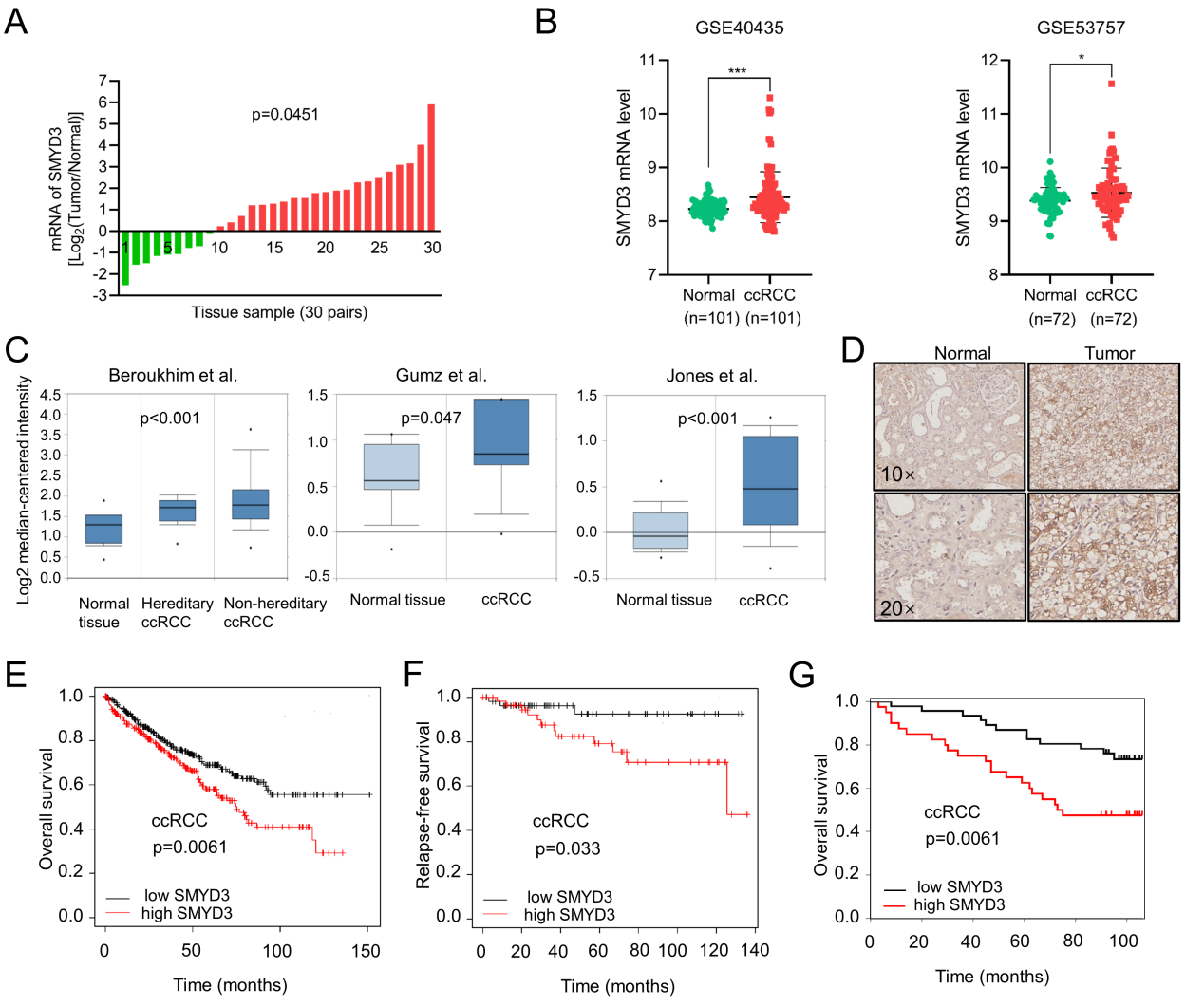
**

**Figure S1.** SMYD3 is upregulated and associated with poor survival in patients with ccRCC: A) mRNA expression of SMYD3 in renal tumor tissues and paired normal tissues (n=30 pairs). B) SMYD3 transcript expression in multiple renal cancer studies from the GEO database. C) SMYD3 transcript expression in multiple renal cancer studies from the Oncomine database. D) Representative IHC staining images of SMYD3 expression at the protein levels in tumors compared with in normal tissues from the TMA cohort. E) and F) Kaplan–Meier survival analysis of overall survival (E) and relapse-free survival (RFS) (F) according to SMYD3 mRNA levels in patients with ccRCC from the KM-plotter database. G) Kaplan–Meier plots comparing the OS of ccRCC patients according to SMYD3 protein abundance in the the TMA cohort. p values were obtained by Student’s t test (A-C) or Log-rank test (E-G), *p < 0.05, ***p < 0.001. Data are presented as mean ± SEM.

**Figure S2**

**
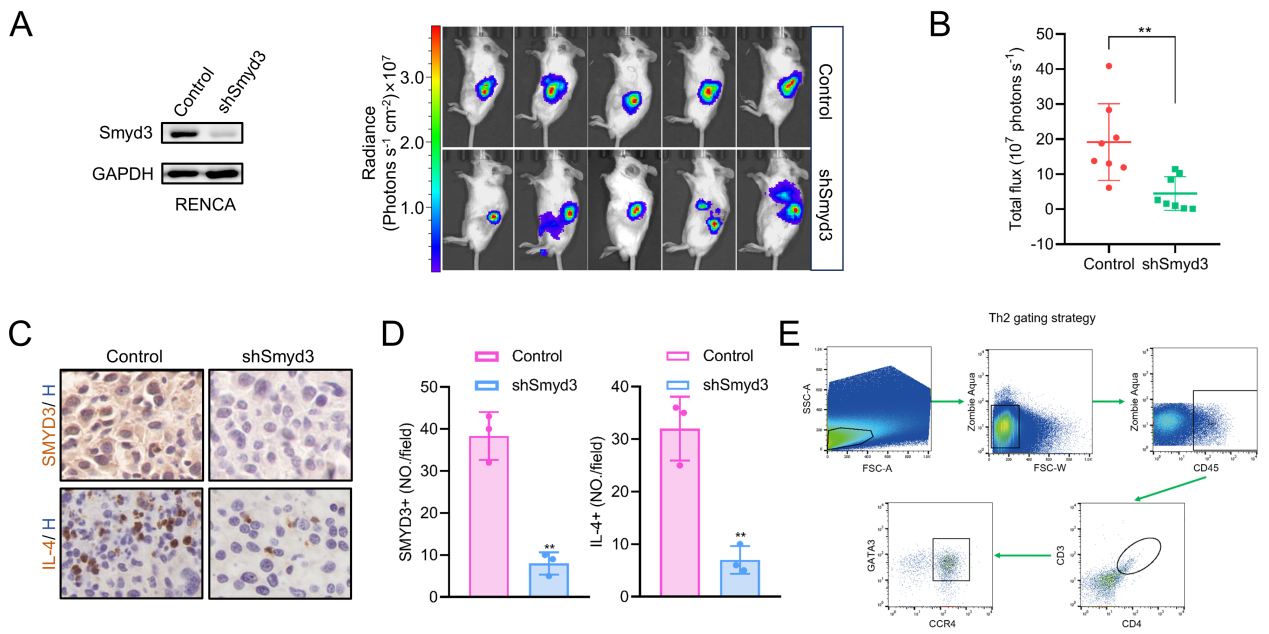
**

**Figure S2.** Smyd3 knockdown impaired tumor growth in the orthotopic syngeneic mouse model *in vivo*: A) Representative bioluminescence images of tumors in Balb/c mice orthotopically engrafted with control or Smyd3-knockdown RENCA tumors. B) Burden of control and Smyd3-knockdown RENCA tumors as measured by bioluminescence (n=8). C) Representative images of orthotopic syngeneic mouse tumors stained for SMYD3 and IL-4. D) Quantification of IHC staining for SMYD3 and IL-4 (n=3). E) Gating strategy of Th2. p values were obtained by Student’s t test (B, C), **p < 0.01. Data are presented as mean ± SEM.

**Figure S3**


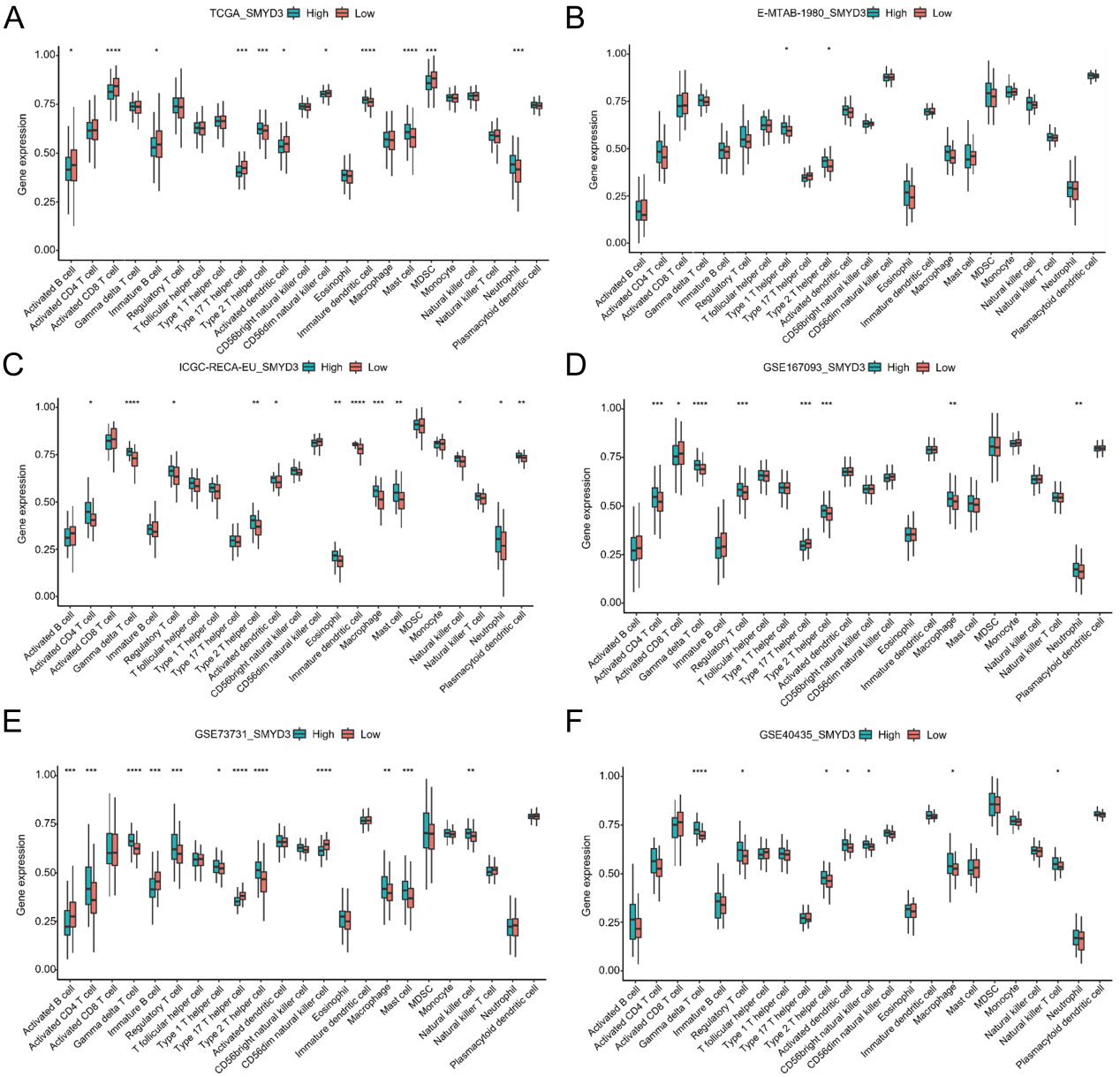


**Figure S3.** Correlation analysis of SMYD3 with immune cell infiltration in ccRCC: A-F) The immune cells infiltration analysis based on SMYD3 mRNA levels by ssGSEA in ccRCC cohorts (TCGA, E-MATAB-1980, ICGC-RECA-EU, GSE167093, GSE73731, and GSE40435). *p < 0.05, **p < 0.01, ***p < 0.001. Data are presented as mean ± SEM.

**Figure S4**


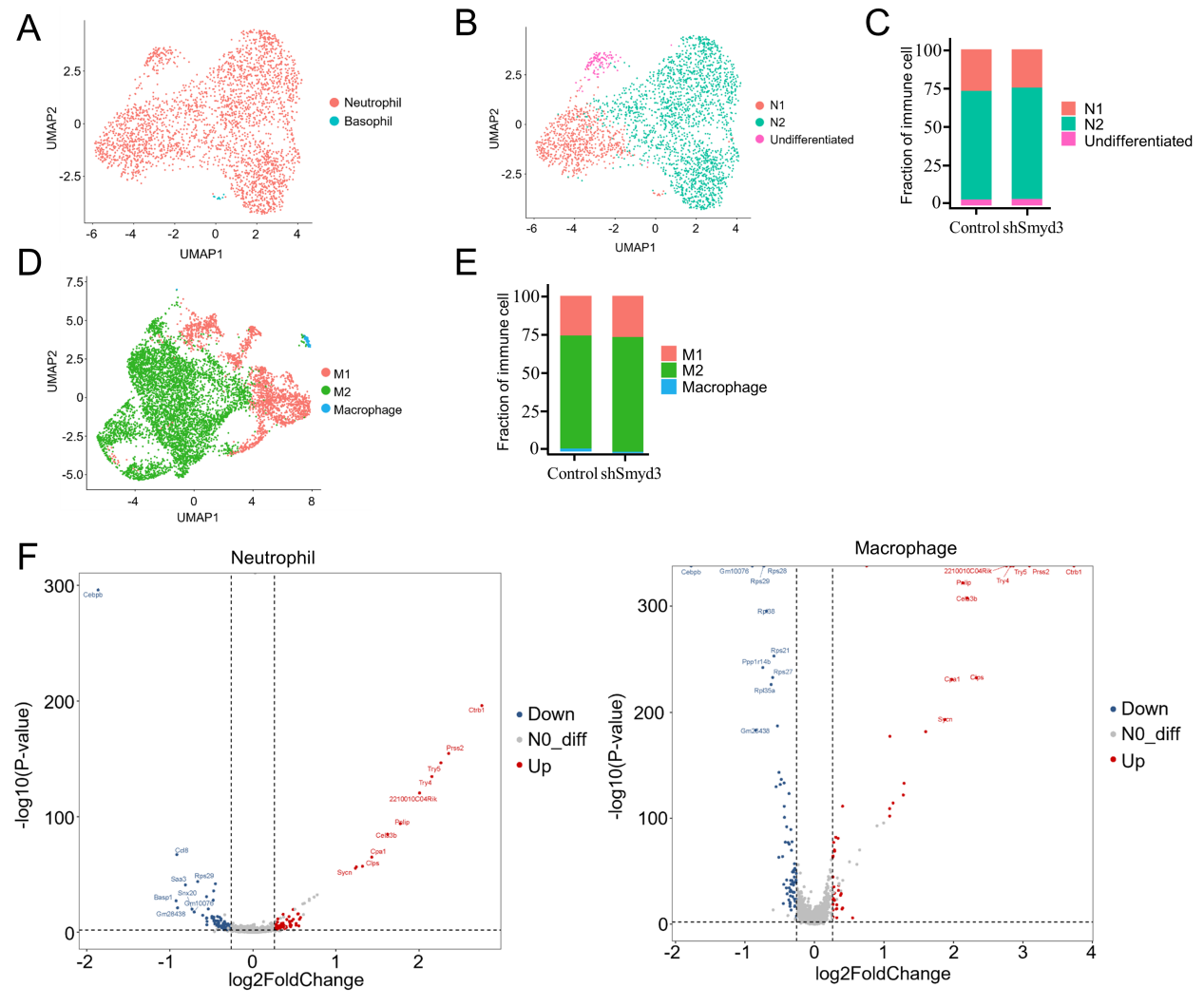


**Figure S4.** Downregulating expression of SMYD3 drove anti-tumor transcriptional changes in the RENCA tumor immune infiltrates. A) UMAP plot of granulocyte showing 2 major clusters identified by scRNA-seq. B）UMAP plot of neutrophil showing 3 major clusters identified by scRNA-seq. C) Bar graph showing the proportions of neutrophil subsets in control and shSmyd3 group. D）UMAP plot of macrophage showing 3 major clusters identified by scRNA-seq. E) Bar graph showing the proportions of macrophage subsets in control and shSmyd3 group. F) Volcano plot of transcriptional changes in neutrophil (left) and macrophage (right) upon Smyd3 knockdown in RENCA tumors.

**Figure S5**


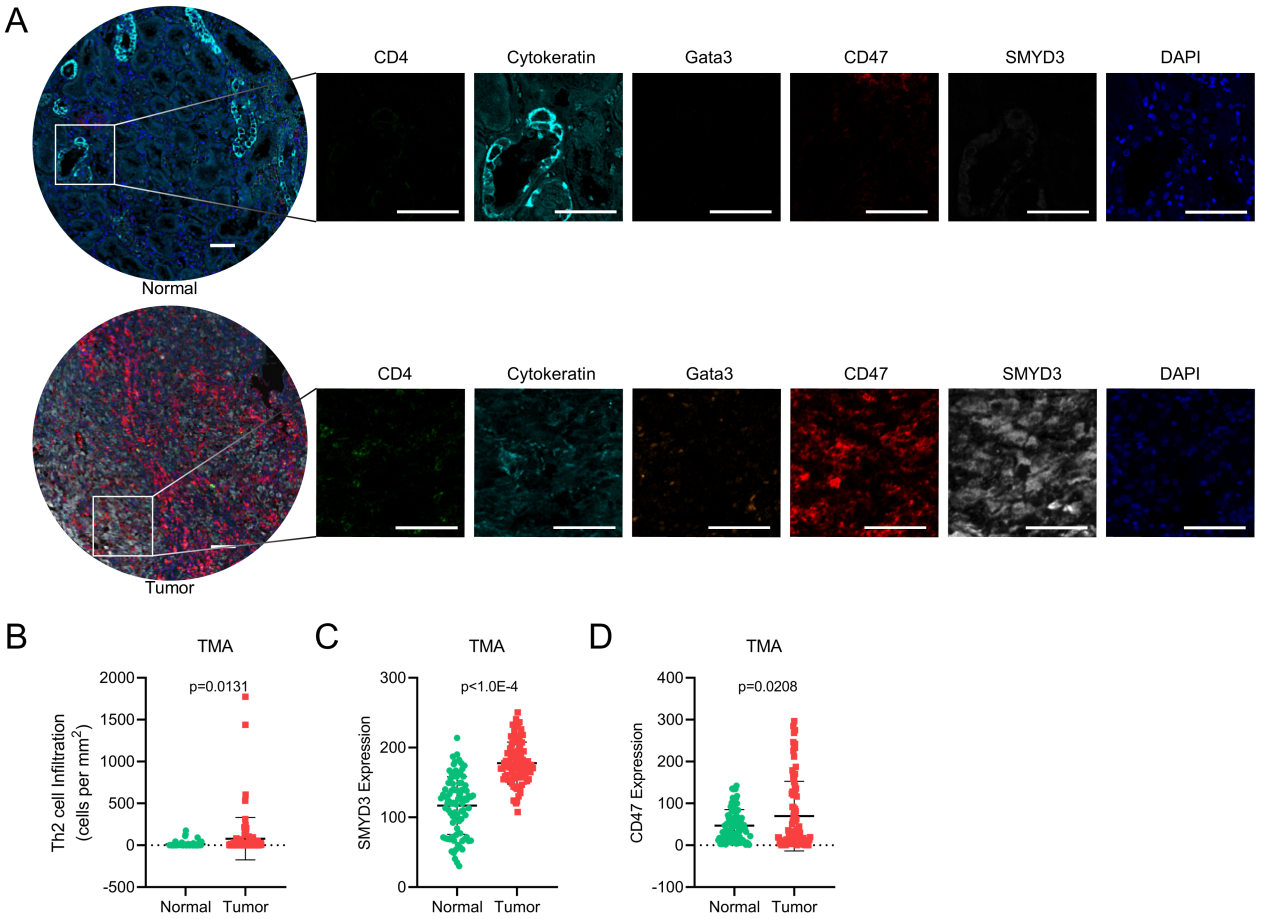


**Figure S5.** The correlation analysis of target protein and Th2 cell infiltration in TMA: A) Representative multiplex immunofluorescence images of renal samples (details given in Table S5) displaying 2 TMA cores after multispectral imaging and enlarged subsections of the core showing each of the individual markers in the composite image after spectral unmixing. Markers: CD4 (Opal 520, pseudocolored green), cytokeratin (Opal 480, pseudocolored cyan), Gata3 (Opal 620, pseudocolored orange), CD47 (Opal 690, pseudocolored red), SMYD3 (Opal 780, pseudocolored white) and DAPI was used as a nuclear marker (pseudocolored blue). Scale bars: 100 μm. B) Quantification of Th2 cell infiltration in the TMA per tissue area (mm^2^) in patients with ccRCC (n=90 pairs). C) and D) The expression of SMYD3 (C) and CD47 (D) in the renal tumor tissues and paired normal tissues in the TMA from the data of multiplex immunofluorescence assays (n=90 pairs). p values were obtained by Student’s t test. Data are presented as mean ± SEM.

**Figure S6**

**
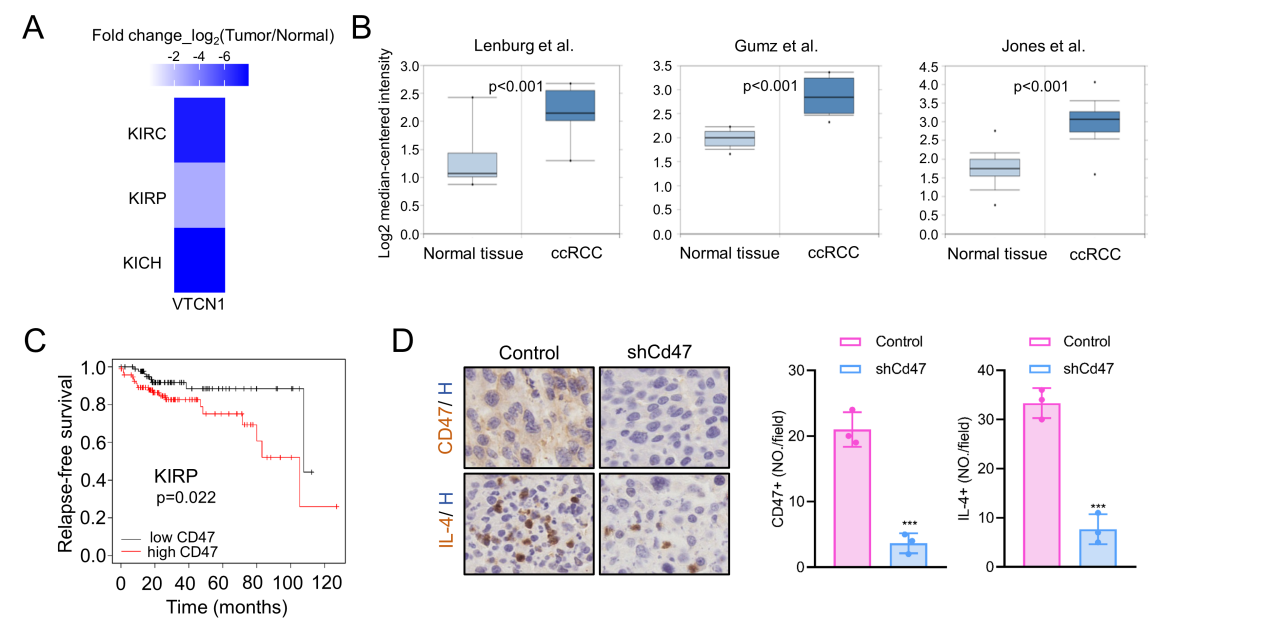
**

**Figure S6.** Upregulated CD47 is positively related to Th2 cell infiltration in ccRCC: A) The fold changes of VTCN1 expression in 3 histologic subtypes of RCC from the TCGA dataset. B) CD47 transcript expression in multiple renal cancer studies from the Oncomine database. C) Kaplan–Meier survival analysis of relapse-free survival according to CD47 mRNA levels in KIRP patients from the KM-plotter database. D) Representative images of orthotopic syngeneic mouse tumors stained for CD47 and IL-4, and the quantification of IHC staining for these two targets (n=3). p values were obtained by Student’s t test (B and D) or log-rank test (C), ***p < 0.001. Data are presented as mean ± SEM.

**Figure S7**

**
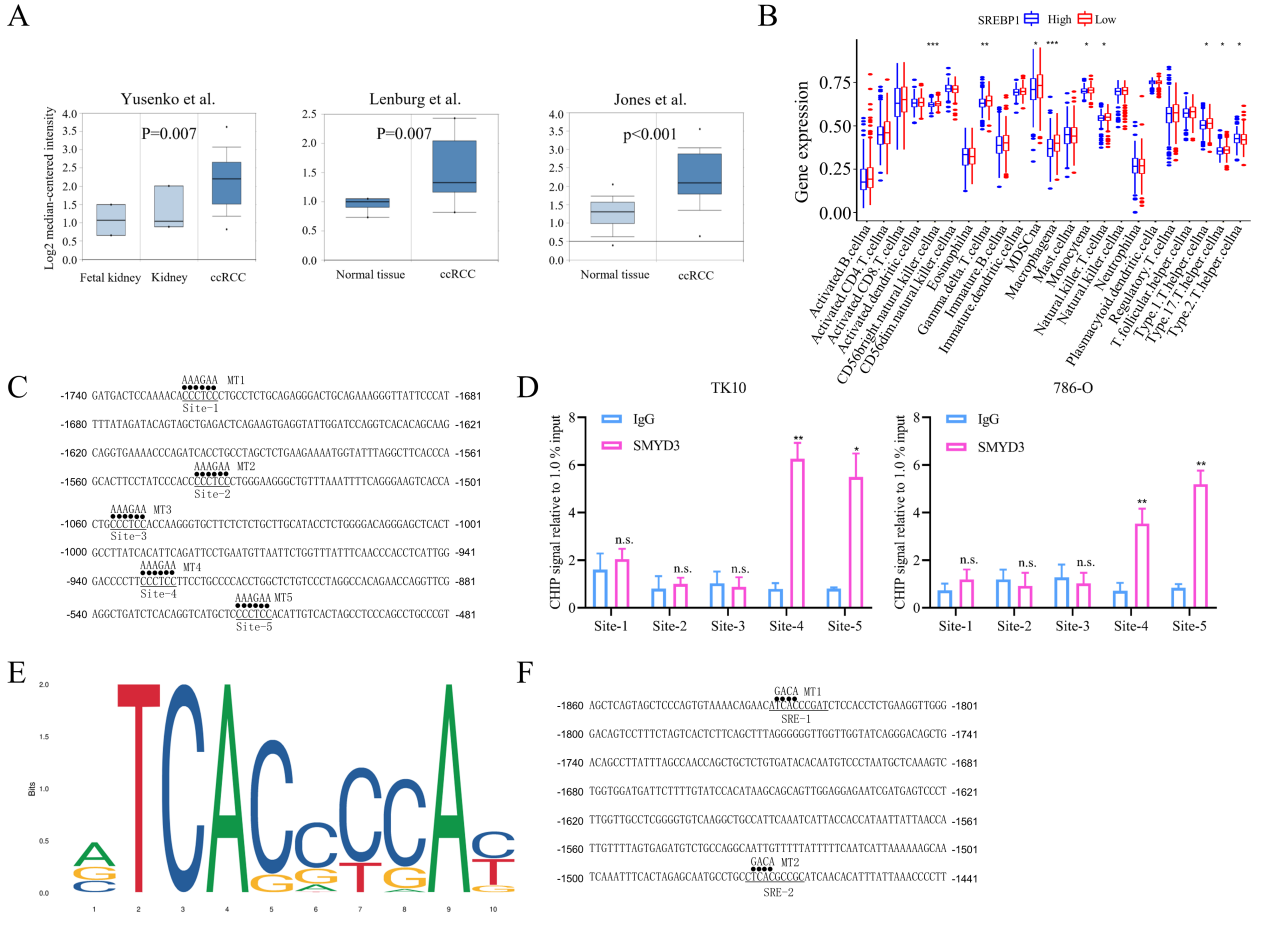
**

**Figure S7.** Upregulated SREBP1 is positively related to Th2 cell infiltration, and the binding sites identified in the promoters of SREBP1 and CD47: A) SREBP1 transcript expression in multiple renal cancer studies from the Oncomine database. B) The immune cells infiltration analysis on the basis of SREBP1 mRNA levels by ssGSEA in TCGA ccRCC dataset. C) Sequence of the SREBP1 promoter region. Five putative SMYD3 binding sites are underlined. The sequence that was mutated in the transcriptional activity analysis of cis-acting elements (MT1–MT5) is indicated by dots, and substitutions are given above. D) Quantitative ChIP assay for SMYD3 occupancy at the SREBP1 promoter (Site1-Site5) in TK10 and 786-O cells (n=3). E) Sequence of SREBP1 motif is provided by the JASPAR database. F) Sequence of the CD47 promoter region. Two putative SREBP1 binding sites are underlined. The sequence that was mutated in the transcriptional activity analysis of cis-acting elements (MT1 and MT2) is indicated by dots, and substitutions are given above. p values were obtained by Student’s t test, n.s., non-significant, *p < 0.05, **p < 0.01, ***p < 0.001. Data are presented as mean ± SEM.

**Figure S8**

**
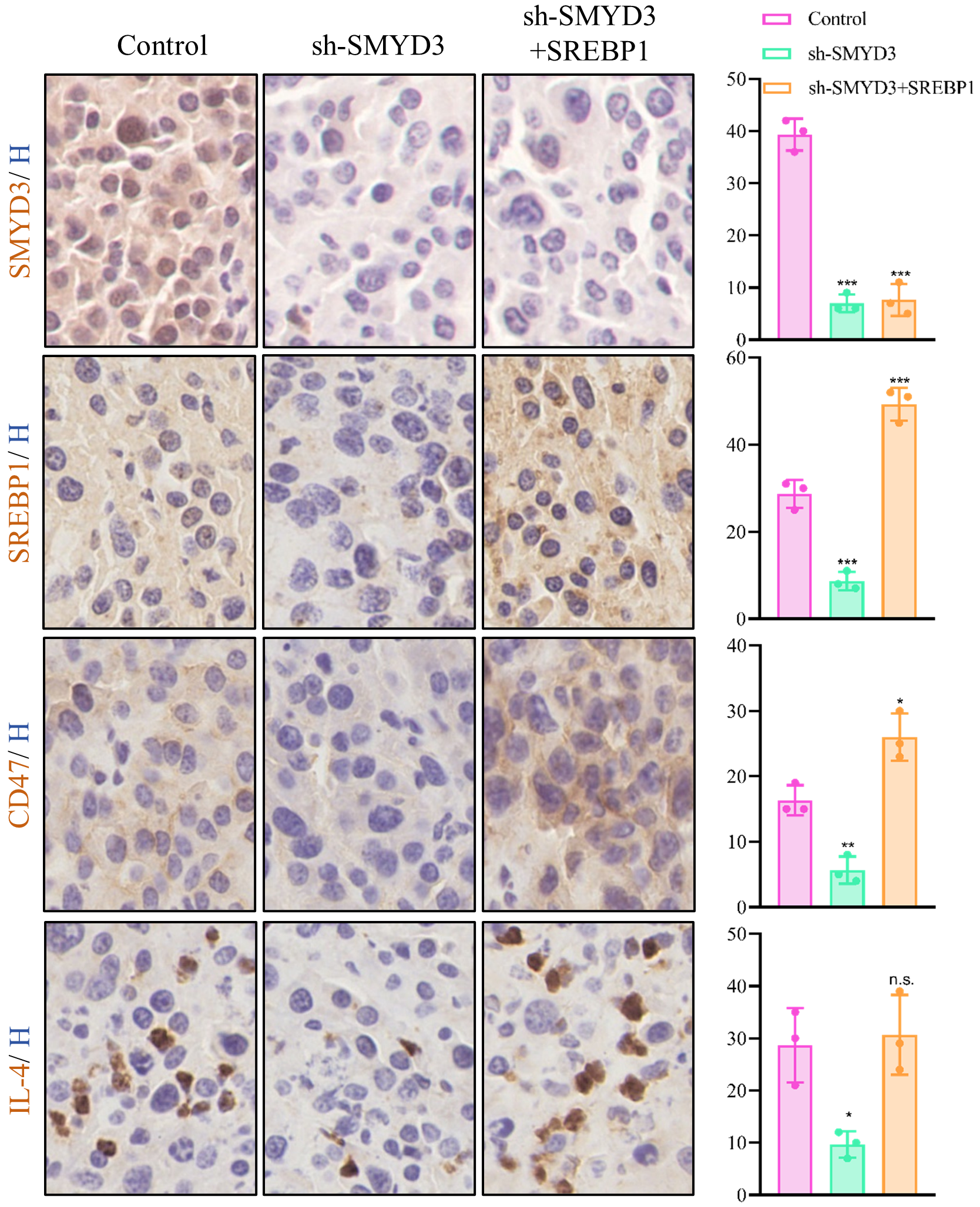
**

**Figure S8.** Representative images and quantification of orthotopic syngeneic mouse tumors stained for SMYD3, SREBP1, CD47, and IL-4. p values were obtained by one-way ANOVA test, n.s., non-significant, *p < 0.05, **p < 0.01, ***p < 0.001. Data are presented as mean ± SEM.

**Figure S9**

**
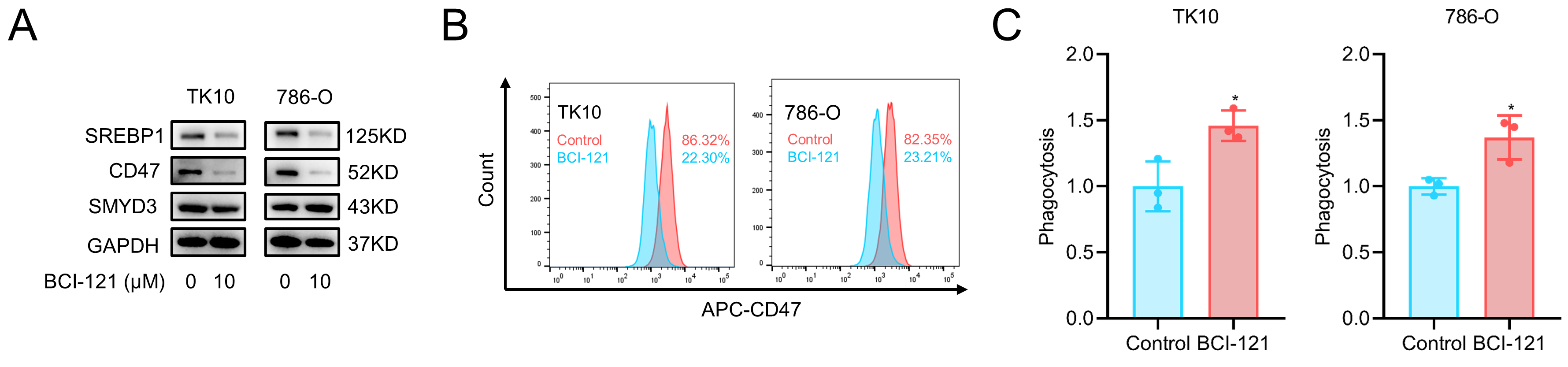
**

**Figure S9.** Effect of BCI-121 on the SREBP‒CD47 axis and phagocytosis: A) The effect of BCI-121 on regulating SREBP1 and CD47 expression was determined by Western blotting and qPCR. B) The CD47 expression on the cell surface was determined by flow cytometry treated with BCI-121. B) TK10 and 786-O cells were exposed to 10 μM BCI-121 for 72 h, labeled with the fluorescent dye CFSE, incubated with human peripheral blood monocyte-derived macrophages for 2 h, stained with CD11b and analyzed by flow cytometry. The phagocytosis rate was calculated as the percentage of CFSE+CD11b+ cells in CSFE+ cells (n=3), and the phagocytosis rate in control cells was set as 100%. p values were obtained by Student’s t test, *p < 0.05. Data are presented as mean ± SEM.

Table S4. characteristics of 30 ccRCC patients with tumors and paired normal tissues

| Age |  |
| --- | --- |
| Median | 62.50 |
| (range) | 38-75 |
| Sex, n (%) |  |
| Male | 21 (70.00) |
| Female | 9 (30.00) |
| AJCC stage^a^, n (%) |  |
| I | 17 (56.67) |
| II | 4 (13.33) |
| III | 6 (20.00) |
| IV | 3 (10.00) |
| T Stage, n (%) |  |
| ＜T2 | 16 (53.33) |
| ≥T2 | 14 (46.67) |
| Lymph node metastasis, n (%) |  |
| Positive | 1 (3.33) |
| Negative | 14 (46.67) |
| Unknown | 15 (50.00) |

^a^Tumor AJCC stages according to the American Joint Committee on Cancer (AJCC) 7th edition.

Table S5. Clinic-pathological data of 90 ccRCC patients contained in tumor tissue microarray (TMA)

| Age |  |
| --- | --- |
| Median | 59 |
| (range) | 29-82 |
| Sex, n (%) |  |
| Male | 59 (65.56) |
| Female | 30 (33.33) |
| Unknown | 1 (1.11) |
| AJCC stage, n (%) |  |
| I | 55 (61.11) |
| II | 24 (26.67) |
| III | 6 (6.67) |
| IV | 2 (2.22) |
| Unknown | 3 (3.33) |
| T Stage, n (%) |  |
| ＜T2 | 57 (63.33) |
| ≥T2 | 33 (36.67) |
| Lymph node metastasis, n (%) |  |
| Positive | 2 (2.22) |
| Negative | 85 (94.44) |
| Unknown | 3 (3.33) |
| M Stage, n (%) |  |
| M0 | 90 (100.00) |
| M1 | 0 (0.00) |

Table S6. Nucleotide sequence of siRNA and shRNA used in this study

Gene Symbol Oligonucleotide sequence (5'->3')

| si-SMYD3#1 (h) | Forward GAU UGA AGA UUU GAU UCU A TT |
| --- | --- |
|  | Reverse UAG AAU CAA AUC UUC AAU C TT |
| si-SMYD3#2 (h) | Forward GCA UCA ACC UCG GCC UGU U TT |
|  | Reverse AAC AGG CCG AGG UUG AUG C TT |
| si-SREBP1#1 (h) | Forward CCU AUU UGA CCC ACC CUA U TT |
|  | Reverse AUA GGG UGG GUC AAA UAG G TT |
| si-SREBP1#2 (h) | Forward CCA UCG ACU ACA UUC GCU U TT |
|  | Reverse AAG CGA AUG UAG UCG AUG G TT |
| sh-SMYD3 (m) | Forward GAT CCG CTG ATG CGT TGT TCT CAA TGT TCA AGA GAC ATT GA G AAC AAC GCA TCA GCT TTT TTA  Reverse CGC GTA AAA AAG CTG ATG CGT TGT TCT CAA TGT CTC TTG AAC ATT GAG AAC AAC GCA TCA GCG |
| sh-CD47 (m) | Forward GAT CCG GAA TGA CCT CTT TCA CCA TTC AAG AGA TGG TGA AAG AGG TCA TTC CTT TTT TA |
|  | Reverse CGC GTA AAA AAG GAA TGA CCT CTT TCA CCA TCT CTT GAA TGG TGA AAG AGG TCA TTC C |

Table S7. Nucleotide sequence of primers used in this study

Gene Symbol Oligonucleotide sequence (5'->3')

| SMYD3 (h) | Forward TGC TGA TGA CCA GTG AGG AGC G |
| --- | --- |
|  | Reverse AAC CTG CTC CCA CTT CCA GTG T |
| SREBP1 (h) | Forward CAC CTG GAC CTG GCT TGT AG |
|  | Reverse CTA GCA TCC ACT CGC AGA GC |
| CD47 (h) | Forward AGA AGG TGA AAC GAT CAT CGA GC |
|  | Reverse CTC ATC CAT ACC ACC GGA TCT |
| CD276 (h) | Forward GCA CAG TTT CAC CGA AGG C |
|  | Reverse AAT CCC GGA TGC TCA CGA AG |
| β-Actin | Forward TGA CGT GGA CAT CCG CAA AG |
|  | Reverse CTG GAA GGT GGA CAG CGA GG |
| SREBP1_site 1 | Forward TGA TGA TGA CTC CAA AAC ACC |
|  | Reverse CAG CTA CTG TAT CTA TAA AAT GGG A |
| SREBP1_site 2 | Forward GCT TCA CC CAG CAC TTC CTA TC |
|  | Reverse CCC TCC AGA TGA ACA ACT GCT |
| SREBP1_site 3 | Forward GGC TTG ACT TAT GAA GGT CTG G |
|  | Reverse TCC CTG TCC CCA GAG GTA TGC |
| SREBP1_site 4 | Forward GTT TAT TTC AAC CCA CCT CAT TGG |
|  | Reverse CCC CAG ATT TGG CAC TTA TTC C |
| SREBP1_site 5  CD47_SRE1  CD47_SRE2 | Forward GCT GAT CTC ACA GGT CAT GCT C |
|  | Reverse AAT GAT GCT TTC TCA CGG GC  Forward ATT CCC AAG AGC AGC AGT GG  Reverse AGA GGT GGA GAT CGG GTG AT  Forward CAG TAG CTC CCA GTG TAA AAC AGA A  Reverse CCC CTA AAG CTG AAG AGT GAC TAG A |
